# The Right to Informed Choice. A Study and Opinion Poll of Women Who Were or Were Not Given the Option of a Sterilisation with Their Caesarean Section

**DOI:** 10.1371/journal.pone.0014776

**Published:** 2011-03-22

**Authors:** Douwe A. Verkuyl, Gerda M. van Goor, Marjo J. Hanssen, Margreet T. Miedema, Marnix Koppe

**Affiliations:** Department of Obstetrics and Gynaecology, Bethesda Ziekenhuis, Hoogeveen, The Netherlands; Institute for Clinical Effectiveness and Health Policy (IECS), Argentina

## Abstract

**Background:**

In the Netherlands, caesarean sections (CSs) are rarely combined with tubal occlusion (TO), partly because discussing CS/TO near delivery is considered unethical and earlier hypothetical counselling – i.e. suppose you happen to need a CS – is rare. This results in more unintended pregnancies and is inconsistent with informed choice. We explored whether TO should indeed not be made routinely available to eligible women.

**Methods and Findings:**

A questionnaire was mailed to 515 Para ≥2 who underwent in the past ≥1 CS. 498 (96.7%) responded. They were on average 35.3 years old, had 2.5 children, had undergone 1.6 CSs, and 3.3 years had passed since their index delivery, either a CS (393) or vaginal birth (105) after a previous CS. 87% of the 498 believed that pregnant mothers with ≥1 children should be routinely counselled about CS/TO. Indeed, 58% and 85% respectively, thought women/couples expecting their second or third child should still be given the TO option days before delivery, if omitted earlier. Counselled women, 138/498 (27.8%), were far more often satisfied than those without CS/TO option. 33/393 had a CS/TO. None indicated regret in the questionnaire. Another 119 also would have elected a CS/TO if given that option. Therefore, 152 (38.7%) of 393 Para ≥2 had or would have liked a concurrent TO. 118/119 wrote they still regretted missing this opportunity. The exception's husband had had a vasectomy. 100/119 were good TO candidates: they were ≥28 years when they delivered an apparently healthy baby of ≥37 weeks. The current contraceptive use of these 100 suggests that this group will have at least 8 unintended pregnancies before age 50.

**Conclusion:**

The experiences and opinions of previous potential candidates for a CS/TO do not support the reluctance of Dutch obstetricians to counsel pregnant Para ≥1 about the TO option for a (potential) CS.

## Introduction

In several European countries, including France, Italy, Austria, Serbia, Greece and the Netherlands, women rarely have their caesarean section (CS) combined with tubal occlusion (TO). However, this CS/TO combination is frequently performed in the United Kingdom (UK), United States (US), Spain, Canada, Ireland, many Latin-American countries, Thailand, Philippines, Sri Lanka, Kenya, Switzerland New Zealand and Australia. For example, a publication from a large hospital in Spain covering the period from 1978 to 1997 (108,776 births) revealed that the proportion of all CSs that were combined with a TO increased from 0.5% to 27.4%, while the CS rate rose from 6.8% to 14.6% [1]. In the Netherlands, the national CS rate was 13.5% in 2002 and we estimate the concurrent TO rate to range from 0.5% to 5%, depending on the obstetrical staff in a specific hospital. In the US, around 13% of all the CS are combined with a TO while the CS rate is more than double that of the Netherlands [2]. This means that both nationally in the US and locally in the Spanish hospital, CS/TO combinations occur 5–50 times more often per delivery than in the catchment areas of the various hospitals in the Netherlands. These large differences between countries and hospitals are also observed within groups of cooperating obstetricians. Thus, for many women in the Netherlands, knowing about and having the option of a TO at CS depends on chance. We should also mention that postpartum (PP) TOs, after vaginal delivery, are extremely rare (about 3 annually) in the Netherlands (191,609 deliveries in 2003). In contrast, in the US (4 million deliveries), about half of the 700,000 annual TOs are performed peripartum: 42% of this half during a CS and 58% PP [2].

Informed patient consent is an important aspect of medical decision taking. It seems unethical and potentially illegal not to counsel the average parous pregnant woman about the CS/TO combination, to give biased information, or to refuse to arrange for a CS/TO if asked to do so [3]. Also, the Charter of the International Planned Parenthood Federation asserts that access to information about reproductive health is a basic human right [4]. Moreover, a widespread consensus is emerging that patients' views are essential to achieving high quality care.

On the other hand many claim that pregnancy is such an emotional state that there is a risk of rash decisions. Also, many obstetricians in rich countries assume problem-free timely access to reliable alternatives later, and there is of course the relative uncertainty concerning the health of the newborn.

There are also many publications that demonstrate increased patient regret after a CS/TO compared to an interval TO, but it is extremely difficult to exclude confounding by (subtle) coercion, and bias related to hospital affiliation and research methodology like case-control studies [5], [6]. Coercion is normally not successful with interval TO, especially not if the patient has to pay herself for that operation. In fact, in many circumstances the psychological, institutional and financial thresholds for interval TOs are so high - requiring strong motivation - that it seems unavoidable that CS/TOs have higher, but not necessarily unacceptable if a substantial number of unintended pregnancies are prevented, regret rates. Crucially, the published CS/TO regret studies did not also examine the regret rates of women/couples who were not given the option of a TO in the event of a CS. This makes it difficult to determine exactly how high the costs are of not counselling women about the possibility of a CS/TO combination. These costs include the number of disappointed women, the expense and complications involved in later having to use another method such as an interval TO, vasectomy, IUD or oral contraception, the occurrence and fear of unintended pregnancies, and the mortality/morbidity related to the unintended pregnancies of women with a scarred uterus. Should these costs be high, restricting access to TO during CS to minimise the frequency of regret may not be reasonable. To ignore the informed consent rule, strong evidence, rather than personal opinion, a routine developed when it was very difficult to obtain an IVF/reversal operation, religious feelings or methodologically often questionable studies[5], [6], is needed.

**Therefore the question we researched was**: Are the views and experiences of the relevant women/couples in agreement with the generally held position of many Dutch obstetricians that in pregnancy an informed choice about the CS/TO option should be withheld?

## Methods

After acquiring ethical permission from the Regional Ethical Review Board *via* the Chairman of the Hospital Board, all 3,678 entries in the Clinical Delivery Register of the district hospital (Bethesda Ziekenhuis) in Hoogeveen in the Netherlands over the period 1 January, 2000 to 1 July, 2006 were scrutinised by the obstetricians & gynaecologists employed by the hospital in 2006. A third of all deliveries in the Netherlands are not “clinical” as these are home deliveries or in-hospital deliveries under the responsibility of midwives, who form the first echelon of maternal care. The national CS rate pertaining to all deliveries was 13.5% in 2002. During the index period, eleven obstetricians with marked differences of opinion about this paper's subject were employed in our hospital long term or for locums.

**Figure 1:**
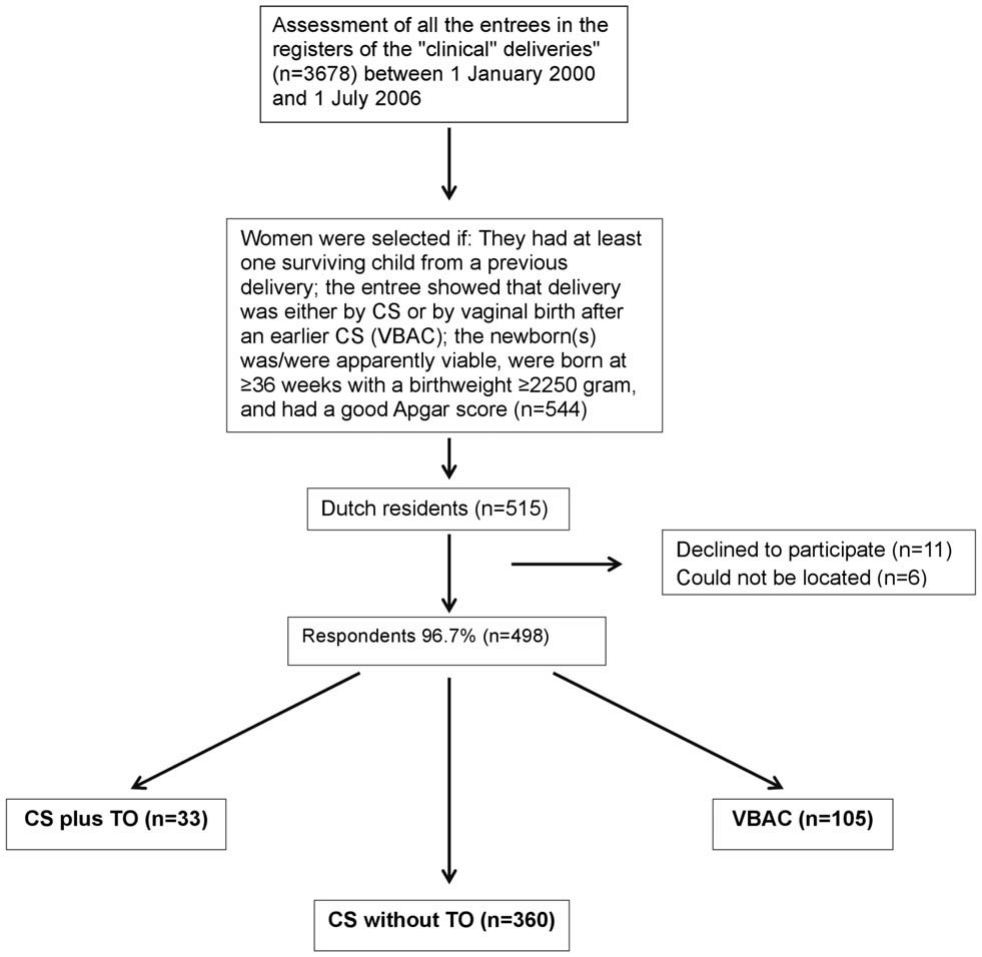
Flowchart. CS = Caesarean section; TO = Tubal occlusion; VBAC = Vaginal birth after earlier CS.

From these 3,678 entries in the register, 544 women were selected, see Flow Chart (Figure 1), because they fulfilled all of the following criteria:

- She had at least one surviving child from a previous delivery.
- Her last, is index, delivery was either by CS, or by vaginal birth after an earlier CS (VBAC).
- The index newborn(s) was/were apparently viable, were born at ≥36 weeks with a birthweight ≥2250 gram, and had a good Apgar score.

Apparently viable in this context means that the delivery notes did not indicate that there were extra concerns just after birth (like worrying congenital abnormalities) about the newborn(s) being at an increased risk of dying.

All 544 women had thus been potential candidates for a CS/TO combination {although a fifth of them ended up having a VBAC and thus a CS/TO even if they were given the TO option was ultimately not available to them}. When women in the registers fulfilled all the selection criteria more than once in the above timeframe, the last delivery was designated the index delivery. 35/544 women had had a CS/TO combination.

After selecting the 544 multipara, 29 expatriate women (two who received a CS/TO) were excluded because their case notes indicated that they were unable to understand more than a few words of Dutch or other European Union language. These women came from many different cultures that varied markedly in their ideal Total Fertility Rates, moreover, translating the questionnaires would often involve difficult to establish conceptual and semantic equivalence. Consequently, even if the answers of these women could have been obtained, they would have been of little relevance for the future management of Dutch/EU residents. These expatriate women came from a refugee centre near the hospital. The political refugee status of some Para ≥4 who had not been offered or were even denied a CS/TO were subsequently not recognised and these women were repatriated with a scar in their uterus to countries with very rudimentary (including lack of access to modern reliable contraception) medical services.

The remaining 515 women, 33 of whom had had a CS/TO, were sent a written introduction (see Supporting Information S1), a questionnaire (see, Supporting Information S2, S3, S4, S5, S6, S7), and a stamped addressed return envelope. We also provided a telephone number and an e-mail address in case clarifications were desired. Women/couples were informed that if they filled in their e-mail address, we would be happy to inform them of the results of the study. In total, 64.5% embraced this option, and this in turn helped us to resolve important omissions or ambiguities in their answers. We emphasised that returning the questionnaire without answers would suffice to show that the addressee did not want to participate and that this would prevent reminders from being sent. The questionnaire contained yes/no, multiple choice and open-ended questions and encouraged extra remarks. The questionnaire was composed with the help of an epidemiologist and a small pilot was staged in another district hospital to help identify and resolve any potential problems. Since there were three main groups of women, namely, those who had a CS/TO, a CS without TO, or a VBAC as last delivery, there were three different questionnaires (see Flow Chart Figure 1, and Supporting Information S2, S3, S4, S5, S6, S7) with those questionnaires in Dutch and translated in English). The answers from the three forms were pooled whenever possible (i.e. the opinions) and the results that were most relevant to our research question are reported here.

This retrospective cohort study could also be defined as an audit combined with an opinion poll.

### Statistical Methods

Statistical analysis was performed after anonymising the data by using Epi-Info version 3.3.2 (2005). Continuous variables were compared by using Student's t-test for normally distributed variables and the Kruskal-Wallis test for non-normally distributed variables. Categorical variables were compared by using the chi-square test or Fisher's exact test.

## Results

In total, 515 women fulfilled our inclusion criteria and were sent the questionnaire. Eleven returned an empty questionnaire and six could ultimately not be located. The enrolment success rate of the study {found + (not always completely) filled-in questionnaire} was 96.7% (n = 498). All 33 women who had had the CS/TO combination participated. The 498 respondents could be subdivided into 393 and 105 women whose index delivery was a CS and VBAC, respectively. Many women wrote long comments and were positively surprised that their opinions were sought. Some of these comments are represented in this paper. Table 1 displays the basic details of the respondents. Table 2 shows that more than half 269/498 (54.%) of them {i.e., 37.6% of the patients who (would have) wanted their CS combined with a TO +16.5% who did not want this but nevertheless would have wanted counselling} had or would have wanted counselling for themselves about the TO option during the index pregnancy. However, 27.7% of all participants had actually received such counselling.

**Table 1:** Basic information about the 498 respondents and their index deliveries.

Variables	CS with TO	CS without TO	VBAC	Total
	N = 33	N = 360	N = 105	N = 498
Mean number of CSs	2.6	1.7	1.0	1.6
Mean number of living children at the time of response	3.4	2.4	2.5	2.5
Mean age at the index delivery in years	34.0	31.8	31.9	32.0
Index CS was elective (%)	31 (93.9)	219 (60.8)	0	250 (50.2)
Last delivery was a failed VBAC attempt (%)	2 (6.1)	65 (18.1)	0	67 (13.5)
Number divorced since the index delivery (%)	1 (3)	14 (3.9)	4 (3.8)	19 (3.8)
Partner has died since the index delivery	0	1	1	2
Had children from a previous relationship (%)	3 (9.1)	14 (3.9)	4 (3.8)	21(4.2)
Number of women who ever lost a live born (the child had appeared viable and mature at birth)	5(1 [last = index baby] died in an accident)	11(1 from congenital heart disease in the first month and 1 from a metabolic abnormality at age 5 [not the index babies])	2(1 from congenital heart disease at age 1.5 and 1 from a metabolic abnormality at the age of 6 months [not the index babies])	18(5 of the total of 1258 children ever born to the 498 women)

TO  =  Tubal occlusion; CS  =  Caesarean section; VBAC  =  Vaginal birth after earlier CS.

**Table 2:** Answers to closed questions/statements relating to the TO option.

Questions/Statements	CS with TO	CS without TO	VBAC	Total
	n = 33	n = 360	n = 105	n = 498
At the time of your last delivery, would you have liked to have your CS combined with sterilisation (if you had delivered by CS)?				
Yes (%)	33 (100)	119 (33.1)*	35 (33.3)**	187 (37.6)***
The doctor did not ask me and I would not have wanted a TO, but I am of the opinion that s/he should have informed me about the TO option.				
Yes (%)	0	74 (20.6)	8 (7.6)	82 (16.5)
I now regret that I did not have a TO at my (possible) CS, but at the time of delivery I was afraid something could happen to my baby and did not want to make an irreversible decision.				
Yes: (%)	0	11 (3.1)	2 (1.9)	13 (2.6)
Who took the initiative to discuss a potential TO with a possible CS?				
Obstetrician	23	47	7	77(15.5%)
You	10	40	9	59(11.8%)
General Practitioner	0	0	0	0
Midwife	0	0	1	1(0.2%)
Nobody	0	272	88	360 (72.3%)
Unclear answer to question	0	1	0	1(0.2%)

TO  =  Tubal occlusion; CS  =  Caesarean section; VBAC  =  Vaginal birth after CS.

*Including three who agreed earlier to a CS/TO, but the TO was not performed/forgotten because of confusion, hurry or marginal prematurity. 20/360 (5.6%) women did not answer or were not sure about their answer.

**Including four who had opted for a TO in the event of a CS, but the TO was not performed because the delivery was vaginal. 4/105 (3.8%) women did not answer or were not sure about their answer.

***24/498 (4.8%) women did not answer or were not sure about their answer.

### Regret about having had or not had a sterilisation

According to their hospital files, two of the 33 women in this study who had had a CS/TO had inquired earlier about reversal. However, by the time they filled in the questionnaire, both were happy they had been sterilised. One had a strong psychosocial indication for the CS/TO, while the other, a grande multipara, had a moderately strong medical indication. Notably, another of the 33 women who received a CS/TO lost her third and last infant in an accident. However, she did not regret having had a TO.

In total, 360 women had a CS without a TO at their last delivery. Of these, 119 (33.1%) wrote on our questionnaire they thought they would have chosen a TO with their CS if they had been offered it (Table 2). Of these 119 women, 118 indicated that they would (at the time of filling in the questionnaire) still be happy with that choice if they would (at the time of their CS) have had a TO. The single exception feared that she would not have felt completely feminine after a TO, her partner obtained a vasectomy.

Of the 119 women, 100 had been - arguably - good candidates for a CS/TO because they were ≥28 years old at the time of their last delivery and their newborns were not even marginally premature. The current contraceptive methods employed by these 100 women and/or their partners are specified in Table 3.

**Table 3:** Contraception in use by respondents; different (overlapping) subgroups.

Method	Women who would have liked a TO with the index CS and were good candidates for it.	Women who thought at the time of delivery that they wanted more children or wanted to maintain that option.	Women who considered, at the time just after the index delivery, their family complete.	“It was my partner's turn to have something done” (Table 5). This is a subgroup of the 309 women in the preceding column.	Contraceptive use of all respondents.
	n = 100	n = 172	n = 309	n = 85	N = 498
Average age at the time of response (years)	36.7	34.0	36.1	35.9	35.3
No contraception	3%	7.6%	1.6%	1.2%	4.6%
OC	27%	38.4%	23.6%	14.1%	28.9%
Condoms	11%	26.2%	11.3%	12.9%	16.7%
IUD, mostly LNG	7%	8.1%	7.1%	3.5%	7.2%
Vasectomy	43% (incl. 2 with LNG IUD and 1 later HYS)	13.4%	38.8% (1 later HYS)	63.5%	29.1% (incl. 3+ LNG IUD, 1+ OC, 1+HYS)
TO	5% (all interval, incl. 2 combined with other operations)	1.2% (both interval)	13.3% (2.6% interval +10.7% CS/TO)	1.2% (interval)	8.6% (10 interval +33 CS/TO)
Implant	0%	0.6%	0.3%	0%	0.4%
NuvaRing®	0%	1.2%	0%	0%	0.4%
Remainder	4%	3.5%	3.9%	3.5%	3.6%

If several methods were used (e.g., condoms plus fertility awareness), the most reliable method was recorded in this table. LNG IUD  =  Levonorgestrel-releasing intra-uterine device; OC  =  Oral contraception; TO  =  Tubal occlusion; CS  =  Caesarean section; VBAC  =  Vaginal birth after CS; HYS  =  post hysterectomy. Remainder: unknown, not at risk, abstinence, lactation amenorrhoea, depot medroxyprogesterone acetate injection, fertility awareness and coitus interruptus.

Of the 105 women who succeeded in a VBAC, 35 (33.5%) would have wanted a TO had they had a CS and been informed of this possibility. This is not dissimilar (p = 0.3) to the 38.7% (152/393) of women who actually had a CS (393) and either had (33) or would have had (119) a CS/TO, if given a choice.

### Whether the CS was long planned or not did not affect the desire for a TO

Whether the CS was elective or not did not much influence the women's desire to have a TO (Table 2), if given a chance. However, as shown in Table 1, it did make a large difference to whether or not the woman could succeed in having this potential desire realised because hypothetical counselling (e.g.: “Suppose you need a CS and de baby seems alright would you then also like a TO?”) was not often performed and then nearly exclusively if there was extra reason to expect a CS. Only two of the 33 CS/TO women had a non-elective CS and both had had a failed attempt at a VBAC (Table 1). Table 2 shows that the TO option had not been discussed during pregnancy with 88 (84%) of the 105 VBAC respondents, despite the fact that the women attempting a VBAC had a 39% (67/172) *a priori* chance of delivering *via* CS.

### Some women do not want a TO to even be mentioned: tact is needed during counselling

Four women were fiercely opposed to the idea of an obstetrician/midwife/general practitioner (GP) starting a discussion about CS/TO, apparently because they thought this insinuated doubt about their fitness as a parent (of a large family) and/or indicated interference in their personal affairs. Moreover, as indicated by the bottom row in Table 4, other women (79 of 486 who answered this question) pointed out, by selecting a multiple choice option, that they personally would not appreciate unsolicited CS/TO counselling in pregnancy because “I am of the opinion that a doctor should not raise the subject of contraception. If I want something or want to know something, I will take the initiative myself”. However, many of these women also qualified their position by writing that women less assertive or informed as themselves could profit from counselling. Many women also warned spontaneously that tact was needed in broaching the TO subject.

**Table 4:** Opinions of respondents about giving pregnant women an informed choice in relation to CS/TO.

Questions	n	Responses
A TO during a CS is easy. Do you think this option should be discussed with a pregnant woman and her partner?:	479	Yes: 418 (87.3%)
If you answered the previous question with Yes, do you think this should be discussed for the first time before the CS for the 2^nd^, 3^rd^, 4^th^, 5^th^, 6^th^, 7^th^, or 8^th^ child (circle the number you prefer)?	479	Mean 2.36(12 respondents wrote “1^st^”)
Do you think that the average Dutch woman is able, together with her partner, **in the last days of her pregnancy**, to make a responsible decision about whether to have a TO combined with her CS?:	471	Yes: 274 (58.2%)
Are you of the opinion that a midwife, obstetrician, or GP should discuss **early during pregnancy** the optionof sterilisation with women who already have children? (*Something like: “suppose you happen to (again) need a CS and a healthy, strong baby is delivered, could you please consider in the months to come whether ou would also like a sterilisation?*) Is such a question appropriate?:	485	Yes: 408* (84.1%)
Consider the example of the enclosed letter involving a woman with 2 children whose third is lying in a transverse position. There is no hurry and the obstetrician **does not** counsel her about the option of a sterilisation with the coming CS. Do you find that:	436	Sensible 17.0%A mistake 74.1%Patronising 8.9%
Consider the example from the enclosed letter involving a woman with 2 children whose third is lying in a transverse position. There is no hurry and the obstetrician **does** counsel her about the option of a sterilisation with the coming CS. Do you find that:	467	Sensible 84.8%A mistake 2.8%Patronising 5.1%Meddlesome 7.3%
I am of the opinion that a doctor should **not** raise the subject of contraception. If I want something or want to know something, I will take the initiative myself: [Indicated “Yes” in different subgroups]	486	Yes: 79 (16.3%)[TO: 28.1%VBAC 12.5%CS without TO 16.3%]

TO  =  Tubal occlusion; CS  =  Caesarean section; VBAC  =  Vaginal birth after CS. *Excluding six women who wrote that a TO should be discussed, but not early in pregnancy.

### Chance of later death of apparently vital newborns

We asked women whether they ever lost a child because this is a well-known reason, beside young age and/or relational strife, for regretting a TO. In total, the 498 respondents had delivered 1,258 live-borns, of whom 18 died before they were 6 years old. Five of these 18 babies died despite having been apparently healthy, (nearly) mature newborns with good Apgar scores (Table 1). This childhood mortality rate of seemingly viable babies (around 1∶250) conforms to the national mortality statistics and the data from the Stichting Perinatale Registratie [7], [8]. The latter foundation also specifies the national perinatal mortality according to gestation and birth weight.

### Men taking responsibility for contraception

Of the women who indicated that they had not wanted (or would not have wanted, given the opportunity) a TO during their (potential) CS (Table 5), 85 selected the following sentence from the multiple choice options: “It was my partner's turn to have something done”. However, 36.5% of these respondents also reported their partner had failed to undergo a vasectomy, thus often requiring the respondents to again shoulder the burden of contraception and/or the increased risk of an unintended pregnancy (Table 3).

**Table 5:** Multiple choice statements selected by women who did not indicate that they had wanted a TO with their last delivery.

Statements selected	CS with-out TO	VBAC	Total
	n = 221	n = 66	n = 287
I want more children.	24 (10.9%)	11 (16.7%)	35 (12.2%)
I want the option of having more children.	112 (50.7%)	35 (53.0%)	147 (51.2%)
I do not want more children, but do not like the idea of not being able to have more.	50 (22.6%)	18 (27.3%)	68 (23.7%)
It was my partner's turn to have something done.	71 (32.1%)	14 (21.2%)	85 (29.6%)
I have little trouble using a reliable method to prevent a pregnancy.	67 (30.3%)	16 (24.2%)	83 (28.9%)
My religion/culture does not allow sterilisation without good medical reason.	1 (0.5%)	0 (0%)	1 (0.3%)

This part was not filled in by 24 women from this group.

For women who had a VBAC, the statements were phrased hypothetically as appropriate. CS  =  Caesarean section; VBAC  =  Vaginal birth after CS.

### Pressure from others to have or not have a TO

The desire for a TO was reported not to be affected by pressure from friends and relatives, nor by religion, the number or gender of the children, a previous divorce, or the earlier loss of a child. Obstetricians introduced the TO option in a neutral or negative (4 times) light. Initiatives by the women/couples were often discouraged (42 times). Women were often told: “We do not like to do that in this hospital/country”, without further explanation.

### Opinions about the need for counselling for CS/TO

Nearly 90% of the women believed that a discussion – preferably in the middle of pregnancy – of the TO option with a possible CS is required. Indeed, 58% and 85% believed that women due to have their second or third child, respectively, should still be counselled about the TO option in the last days before a CS is performed had she not been informed earlier (Table 4).

### The factors preventing the women who would have wanted a CS/TO from having one

As mentioned above, there were exactly 100 women who would - given the option - have wanted a TO with their CS, and who were also good candidates for a CS/TO but who did not receive a TO. These 100 women include two whose request for a TO was forgotten/“forgotten” during the CS. Another 31 women asked for a TO, but their request was not granted. However, two of these women postponed asking for a TO until they were en route to the operating theatre. It should be noted that these two also answered in the affirmative to the option: “I am of the opinion that a doctor should not raise the subject of contraception. If I want something or want to know something, I will take the initiative myself” (Table 4). This suggests that routine counselling could also meet the needs of even those women who feel that it should be their responsibility to inform themselves about the possibility of a TO with a CS. In another two cases, the obstetrician mentioned, but then dismissed the option. In two other cases, the TO option was only mentioned for the first time by the obstetrician minutes before the CS and during the CS, respectively. For 60 women, the subject was not raised at all and at least 33 of these women did not know the possibility even existed. With regard to the remaining three women, it is unclear what happened exactly.

### Potential dissatisfaction had everyone had access to the CS/TO option

In total, 154 (119+35) respondents (Table 2) from the CS and VBAC groups together would have wanted a TO during the index delivery but did not receive a TO, either because of reasons like those mentioned above or because they gave birth vaginally (35 women). Three of these 154 (1.9%, or better 1.6% if the women who actually had a CS/TO are included (3/187)) may have later regretted the TO had they received one. One of these was a mother of three living children during our survey. She wrote that had she and her husband been given the option of a TO (they were not) and had the last delivery been a CS (it was a VBAC), she would have probably chosen a TO. However, her partner died unexpectedly some time after the last delivery. She speculated, 4 years after the death of her partner, that had she found another suitable spouse (she had not), she might have been unhappy that she had chosen a TO. Another woman wrote that she had delivered her first baby by an elective CS due to breech presentation. The operation had been such a bad experience that she dreaded a repeat CS. She said that had she been offered a TO in the next pregnancy, she probably would have accepted and may have regretted that later if the second CS had not been as unpleasant as the first one. However, the second delivery was a VBAC. The third woman initiated a discussion with the obstetrician about having a TO during her elective CS for her second (twin) pregnancy. Her first delivery had also been a CS. She did not have the TO after receiving negative advice from the obstetrician. She is also the woman mentioned above who indicated that a TO might have made her feel less feminine and whose partner had instead a vasectomy. Nevertheless, all three of these women were still of the opinion that pregnant women should be informed about the TO option, although the latter two specified this information should only be provided in the form of a pamphlet.

### The likelihood that women have an interval TO later

Five of the 100 good candidates for CS/TO who regretted having missed the TO opportunity, had a TO later. Frequently the obstetrician had turned down or discouraged a request for a CS/TO combination while promising instead to perform an interval TO at a later date. However, some women discovered later that this option was too expensive (Table 6) and at least four women were told later that they were not suitable for a laparoscopic TO because of health concerns. Overall, 10% of the 360 women who had a CS without a TO were according to their case notes poor candidates for a laparoscopic TO later because of adhesions seen at CS, severe obesity, an incisional hernia after an infection, or an earlier thrombotic event. Other reasons for not having an interval TO are listed in Table 6.

**Table 6:** Multiple choice statements selected by women who had indicated that they would have wanted a TO with the index delivery but who did not receive one.

Statements selected	CS with- out TO	VBAC	Total
	n = 119	n = 35	n = 154
I do not want to have more children and feel burdened by having to arrange contraception.	46 (38.7%)	18 (51.4%)	64 (41.6%)
It would have been convenient to have had a TO with my CS, but my partner and I have little trouble using reliable contraception.	33 (27.7%)	22 (62.9%)	55 (35.7%)
I worry that I will become pregnant by accident one of these days.	23 (19.3%)	4 (11.4%)	27 (17.5%)
I would like a TO now but am afraid of the operation.	22 (18.5%)	9 (25.7%)	31 (20.1%)
I would like a TO now but procrastinate organising it.	14 (11.8%)	4 (11.4%)	18 (11.7%)
An interval TO is too expensive.	4 (3.4%)	3 (8.6%)	7 (4.5%)
I think my partner and I lacked foresight when we didn't take the initiative to get a TO with the CS. It was a missed opportunity.	29 (24.4%)	3 (8.6%)	32 (20.8%)
I asked for a TO but the obstetrician advised against it/refused.	36 (30.3%)	6 (17.1%)	42 (27.3%)
There was actually a good medical reason for a TO.	26 (21.8%)	2 (5.7%)	28 (18.2%)
The obstetrician raised the subject of having a TO with the CS but dissuaded me at the same time. This, regrettably, decided me against having a TO.	4 (3.4%)	0 (0%)	4 (2.6%)

For women who had a VBAC, the statements were phrased hypothetically as appropriate. TO  =  Tubal occlusion; CS  =  Caesarean section; VBAC  =  Vaginal birth after CS.

## Discussion

### Similar studies which compare having a choice with not having a choice

As far as we know, there is only one publication that compares the views of all relevant women, namely, women above a defined age with a specified minimum number of children who delivered by CS with and without the option of having a concomitant TO [9]. This retrospective cohort study was performed in Zimbabwe and involved 784 successfully interviewed women, 553 of whom had a TO during CS. The study revealed that women who were not given the option of a TO before undergoing an elective or emergency CS (n = 137) were 8.8 times more likely to be dissatisfied at follow-up (64.2%, 88/137) than women who were offered a TO (n = 647, dissatisfaction rate 47/647 = 7.3%). Moreover, of the 47 women offered a TO who were not happy later, most (n = 31, 66%) were dissatisfied because they had not taken that option. The remaining 16 (16/647 = 2.5%) dissatisfied women were unhappy because they had agreed to the TO. However, only three of these women were interested in the offer of an all-costs-paid reversal operation, of which one, after HIV tests, was ultimately performed. Women who had an emergency CS regretted the TO less often, but had on average 1.3 more children, than those with an elective (mostly repeat) CS (mean 4.4 children).

The above study needed to be repeated in Europe because the situation there is so dramatically different. For European women, a permanent method may be more appropriate because they have a smaller ideal family size – the actual Total Fertility Rate is 1.7 in the Netherlands – than women in sub-Saharan Africa and they live in an environment where child mortality is 25 times lower. On the other hand, limiting their access to a TO has less severe consequences than in sub-Saharan Africa because they have easier access to modern and reliable alternatives (including vasectomies for their partners) and safe abortions. Unmet need for contraception is widespread in under-resourced countries [10]. Demographic and Health Surveys in 27 developing countries indicate that two-thirds of postpartum women had unmet needs for contraception [11]. Although European women often complete their families (with frequently exactly the number of children they wanted) with fewer children than their sub-Saharan counterparts (with often 1–2 children more than originally seen as ideal) this achievement occurs typically at around the same age: e.g. 2 and 5 children respectively, both approximately at age 32. The former women have by that time many years of experience in avoiding pregnancies with reversible methods, the latter mostly not. This makes women in Europe probably better placed to prevent unintended pregnancies for the many fertile years still to come without resorting to TO. Moreover, the chance of a European woman dying in a subsequent pregnancy is very low, unlike many women in sub-Saharan Africa, whose chance of dying in a subsequent pregnancy exceeds 1% if they have a uterine scar [12], [13]. In remote areas of developing countries ante-natal care is often minimal and therefore mid-pregnancy counselling of multipara without a history of CS, about a possible TO with a potential emergency CS, usually performed by a doctor or medical assistant never seen before, is rare. While in this group of women above all a scar in the uterus might easily have fatal consequences.

Indeed, the current situation in, for example, Somalia, Ethiopia, much of Sudan, Tchad, Congo, Cote d'Ivoire, Niger and also Afghanistan, means that fertile women in these areas who have had a previous CS are in extreme danger. In fact, this applies to all countries where vesico-vaginal fistulas caused by obstructed labour (global, annual incidence 50.000–100.000; WHO) are still prevalent. Therefore, it could well be that multipara in these areas who had a TO during a CS or after admission following an unsafe abortion are less likely to regret a combined TO (if they had been given the option) than to die of the next pregnancy (if they did not have or use the option) [9]. Conversely, African women have few employment opportunities and little social security or pension provisions. Consequently, if they lose their partners (in recent times, often because of HIV or civil unrest related to population pressure), they are more or less forced to start a new relationship, which is often expected to be cemented with at least one child. This may lead to higher rates of regret after a CS/TO. In addition, the possibility that patients with severe regret will receive reversal operations or *in vitro* fertilisation (IVF), interventions for which the institutional thresholds are presently low in many European countries including the Netherlands is usually remote in Africa. The above considerations underline the importance of also determining the views about CS/TO counselling held by European residents. For this reason, we sent a questionnaire to 515 women in the Netherlands who had previously been potentially eligible for CS/TO.

### Regret about having been sterilised and about not having had that option

None of the 33 women who had a CS/TO combination were dissatisfied at our survey. In contrast, 119/360 (33.1%) of those who had a CS without TO (Table 2) indicated they would have chosen that combination if given the opportunity, and all but one of these, 118, still felt regret when they responded, that they had not been granted that option. Thus, of the 393 Para ≥2 whose index delivery was a CS, 152 (38.7%) would have wanted a CS/TO combination, but only 33/152 (8.4% of the 393) actually succeeded in having one. As far as we can ascertain from the responses to the hypothetical questions in the questionnaire, only one of these 152 women might have later regretted having chosen a TO had she been provided with one (although she did not desire any further pregnancies). Had all 152 TO-desiring women actually been given a TO during their last CS, this would have increased the CS/TO combination rate relative to the deliveries in the hospital's catchment area from 0.6% to 2.8%. {The “clinical” frequencies of respectively 0.9% and 4.2% actually calculated by us were adjusted to 0.6% and 2.8% to compensate for deliveries under the responsibility of first-line midwives.}

The 2.8% is approaching the rate of 3.9% in the Spanish hospital that was described in the Introduction (this rate was relative to all deliveries over the last 5 years) [1]. Thus, the desire for a TO of the women in the two different European countries seems more similar than the actual frequency with which CS/TOs are provided, which suggests that it is the different traditions and opinions of obstetricians that are responsible for the differences in the actual CS/TO rates.

Hypothetically, if all the 498 participants (393 CS +105 VBAC) would have delivered by CS and all would have had in time the option of a TO as is the practice of some obstetricians, probably 1–3 (0.2%–0.6%, see one but last sub-chapter of “Results”) of them would have regretted the outcome as a direct result of having had the TO option. If none would have been given the option, as is the practice of some other obstetricians, 33+118+35 = 186 (37.3%) would have had regrets as a direct result of not having had the TO option. Therefore, we have some evidence, that a policy of not giving the patient/couple an informed choice is in our practice 62 to 186 times more likely to disappoint.

### Contraceptive failure in the women who would have wanted a TO during CS but did not receive it

100 of the 119 women who had a CS and would have wanted, but did not receive, a concomitant TO were very good candidates for the operation. At the time of our survey, 5 and 43 of these prevented conception by an interval TO and by vasectomy, respectively (Table 3). With regard to the remaining 52 women, should they continue using the contraceptive methods they were using at the time of our survey until their 50^th^ birthday, this would equate to 346 years of oral contraception, 6 years of contraceptive injections, 134 years of condom use, 96 years of intra-uterine device (IUD) use, and 27 years of coitus interruptus. Considering the typical-use failure rates of these methods [2], [14], and dividing those arbitrarily by 5 to compensate for aging, the 52 women could expect to have between them eight to ten unintended pregnancies [14], [2]. Indeed, at least two unintended pregnancies have already occurred in the on average 3.8 years (380 years of observation) between these 100 index deliveries and our survey. We know (after further opportunistic observation up to the end of 2007) of another originally unintended birth in this group of hundred. Furthermore, we were informed about one more unintended pregnancy in the group of 6 women we could not locate, see Flow Chart. We approached her family to ask for her new address. But then she let us know via her father, that she refused to cooperate since she was very angry with us because she was denied a TO with her index CS and delivered again - elsewhere - within a year.

### Unplanned third and fourth pregnancies occur often

A survey in the Netherlands (which, of the countries with reliable relevant official records, has one of the lowest rates of induced abortion) showed that resident women who had completed their families accounted for 36% of all unintended (aborted or not aborted) pregnancies, this proportion trebled over the preceding 20 years [15]. Similarly, in Denmark in 2001, women with induced abortions were 2.8 times more likely to have ≥2 children than 1 child {30.9% *vs* 11.1%} [16]. In 2008 in the Netherlands these figures were 1.5 times and 30.5% *vs* 20.6% respectively [17]. Analyses of the data of 6 Dutch abortion clinics which use the same software for data registration show that of the 14,199 women who had abortions there in 2010, 32.2% gave a “completed family” and/or “being too old” as reason. In England and Wales among women seeking an induced abortion older women are significantly (p<0.01) less likely to use a regular method of contraception [18].

It should be noted that pregnancies including spontaneous miscarriages often will not be confessed as unintended to an investigator or even friends and family and will consequently not appear in any statistics. However, obstetricians and midwives often hear such stories in the privacy of their office (if they inquire). Aborted unintended pregnancies are of course easier to quantify in most countries where they are legal. Our unpublished study in an abortion clinic in Leiden showed that of 546 Dutch residents who were ≥36 years, had ≥2 children, and aborted there in 2003–2006, 43 (7.9%) had delivered the last time by CS. The records typically show that such women have children of school age and have restarted their careers, and that their unintended pregnancies compel them to make an unsettling decision. Thus, had these women been given the option of a CS/TO, at least some of them would have been spared from having to make this painful choice. The obstetrician involved in the last CS seldom would hear about these induced abortions but, conversely, would nearly always be informed if a patient regretted a CS/TO or about the rare failed TO. This feed-back bias probably affects policy.

### Failure rates of TO and the efficacy of its alternatives

Numerous papers have shown that (medicated) IUDs *in situ* are as reliable as peripartum TOs and have of course the added advantage that their effect is easily reversible [2], [19], [20]. This fact is often mentioned, together with their non-contraceptive benefits in the case of medicated IUDs, against the CS/TO combination. However, it is important to emphasise that these IUD studies do not reflect the real usefulness of IUDs in this situation because they are never reported on an intention-to-use basis: as a result, these papers do not register the pregnancies due to postponements or cancellations in the decision to fit an IUD or in its actual insertion. Such postponements and cancellations arise for a number of reasons, including: the clinic/GP staff typically (often mistakenly) instruct women to wait until their menses have arrived; protocol demands that Chlamydia tests are performed first; GPs are reluctant to insert an IUD in a uterus with a fresh scar; an alternative healer is consulted and advises against all hormones, the woman may have to pay for the insertion of an IUD; the woman may feel anxious about the procedure; there are waiting lists for such procedures; and the woman becomes pregnant before her first postpartum visit [21], [22], [23]. An intended CS/TO combination, although the obstetrician sometimes forgets to perform the TO, is not subject to such delays and mistakes. Moreover, IUDs are often removed because of side-effects. The guidelines of the UK National Institute for Health and Clinical Excellence (NICE), advises that healthcare professionals should know that up to 60% of women (although probably disproportionally those whose families are not yet completed {authors}) discontinues using medicated IUD within 5 years because of pain, irregular bleeding and/or systemic progestogenic adverse effects [24]. The IUD is then often replaced by less effective methods. Notably, the age of the subjects participating in our study means they often would need to be fitted with three or four medicated IUDs before ovulation becomes very unlikely. Obstetricians/gynaecologists in the Netherlands insert far more IUDs than they remove. This is often done by a GP. Another feed-back bias.

Therefore, intended CS/TOs for women delivered by CS are associated with much lower actual pregnancy rates till menopause than the intention to use any contraceptive method (including vasectomy, (hysteroscopic) TO and IUD) to be applied after discharge from hospital following a CS.

It should be noted here, as an aside, that we believe IUDs, vasectomies or implants are often superior to alternative methods in other situations. For example, not offering the option of an IUD to be placed immediately after an induced abortion in favour of patient's (vague) intention to organise herself an interval TO or IUD later is, in our opinion, comparable to not counselling women about a CS/TO combination in favour of (vaguely) organising an IUD, vasectomy or TO later [21], [25], [26].

Laparoscopic/hysteroscopic TOs and vasectomies are subject to similar errors and delays that typically reduce the effectiveness of intended IUD use compared to that of intended CS/TO combinations [21]. Delays in obtaining interval TOs and IUDs are also increasing because there are presently vacancies for gynaecologists and GPs especially in the Northern areas of the Netherlands. Paradoxically, the relevant politicians have just (in 2010) decided that the economical situation demands the removal of contraceptives from the basic health insurance plan for those over 20 years of age [27], [28]. Worrying is that in the USA the wholesale price of medicated IUDs has increased with 43% from $586 to $843 in 2010 [29]. They presently retail for €139 in the Netherlands, insertion and check up included: €287.

But, on the other hand, at least there are keen commercial interests – hysteroscopic TO for example costs at a minimum €1425 in the Netherlands - stimulating the use of most of the non CS/TO methods. Possibly one of the reasons that CS/TO is so seldom performed is that it is so inexpensive and a satisfied client will not need to pay for contraception again. There is no lobby. If routine CS/TO counselling of pregnant Para ≥1 would be introduced nationally and the option realised as often as our respondents wrote they would have liked, then 3–4% of all women with a completed family would have this very economical form of contraception.

With regard to other contraceptive methods a third of the abortion clients in the Netherlands were (supposed to be) using oral contraception [17]. A study involving a representative (although therefore younger and more fertile than women with a completed family) sample of 7643 women from the US (where IUDs are not used frequently) revealed that reversible contraception is associated with a 12.4% overall failure rate within the first year [30]. In contrast, the prospective US CREST study found that for women who were sterilised peripartum, the failure rate was 7.5 per 1000 procedures cumulative over ten years [2], [19], [31]. Thus, peripartum sterilisation is more than 100 times less likely to fail than reversible contraceptive methods as they are used in the US. It should also be pointed out that half of the 3.1 million annual unintended pregnancies, of which 1.2 million end in an induced abortion, arise in the US because the woman was not using any contraceptive method [32]. Many of those conceptions may have arisen because an opportunity to provide a reliable method like an IUD, vasectomy or TO was missed [33]. Indeed, in the US, about two-thirds of the CSs performed on parous women are not combined with a TO. Two US studies have shown that desired peripartum TOs are frequently not performed because of avoidable (administrative) mistakes/complications [25], [33]. To quote from the latter paper originating from Chicago: “One fears that women who have difficulty negotiating the Medicaid consent process (for a peripartum TO {authors}) may be further challenged in obtaining interval sterilization. This issue may be particularly relevant for the many women who lose Medicaid coverage after pregnancy.”

### Coercion as reason for regret

Many studies have shown that women report regret more frequently after CS/TO than after interval TO, which has made obstetricians in some countries wary about even informing women about the CS/TO combination. However, these studies are often based on the previous generation of women, who received a CS/TO at a time when doctors were more able and inclined to decide for their patients. The women of that generation also tended to be younger when they, people in their environment or the doctor thought that their family was complete, they were less likely to have a career, serious congenital abnormalities were more often missed during pregnancy, childhood mortality was higher and IVF more problematic. Frequent regret seems also unavoidable in some situations where patient education/counselling appears very poor [34]. However, a study from Sweden (culturally not unlike the Netherlands) and a recent study from Brazil did not find that women regretted TO more frequently when performed peripartum [35], [36]. Nevertheless, it is possible that the current generation of women are also subject to coercion: it is technically very easy to have a TO if the abdomen has to be opened anyway. In addition, the CS/TO procedure is not associated with any extra effort, pain, anxiety, risks or costs on the woman's part, and there are also, just like with some other methods, non-contraceptive benefits [37], [38].These factors can be enticing and should lead to extra careful counselling. However, not allowing women to choose whether they want a TO with their CS is also a form of coercion.

It should be mentioned as an aside that the available literature suggests that, unfortunately, members of those social groups who are most likely to regret a TO are also the ones who are most likely to have unintended pregnancies without a TO [2], [19], [34], [39], [40]. Moreover, as mentioned above there exist in some settings much misinformation and many myths concerning TO which have to result in confounding the relationship between the timing of a TO and the frequency of regret [34].

Apart from that, women who have had serious contacts with the child protection authorities or who are victims of intimate partner violence or who are substance abusers or HIV infected are sometimes put under subtle or less subtle pressure when they need a CS to combine it with a TO. This will result in higher regret rates, compared with interval TO, when the entire CS/TO cohort is used as denominator. These data, presented without the specific circumstances of the women involved, in turn will result in a reluctance to offer TO to many women who deliver by CS even if there are no particular risk factors for regret.

### The dilemma and economics

If the serious (requesting reversal) regret rate with interval TO would be 1% and with CS/TO after good counselling twice as high, would that be a reason to deny the 98% a convenient TO? It is difficult to equate the emotional costs of regretted TOs with the emotional costs of (the fear of) unintended pregnancies. Determining these costs is also very personal. For example, women and/or health workers who feel that induced abortions are interventions that should not be taken lightly are likely to feel that unintended pregnancies are more costly than those who have fewer ethical problems with abortion or even see the procedure as just another family-planning method. We acknowledge the stress, effort and expenses related to a regretted TO. However, our study has made it clear that women are far more frequently dissatisfied if their right to informed autonomy is not respected. Furthermore, the provision of the CS/TO option seems the more economical choice.

It may therefore be appropriate for governments and/or insurers to consider facilitating permanent contraception when the opportunity arises, while simultaneously committing themselves to pay for *in vitro* fertilisation/intracytoplasmic sperm injection (IVF/ICSI) or reversal operation if a partner or child dies later. As an example, this happened after a serious earthquake in China[41]. We estimate that, should eligible women in the Netherlands be granted a CS/TO combination as often as the women participating in this study would have liked, and that this would be followed by an improbable requested reversal rate as high as 2% [42], then nationally eighty women would request reversal/IVF annually. This 2% rate seems high in the light of our results en also because “only” 2.4% of the men in the Netherlands who undergo a vasectomy have a reversal operation within 10 years [43]. Men are likely to have higher regret rates than women because men can still socially acceptable [44] (often with another partner) and biologically feasible realise the desire for more children when they are older than 40 years and furthermore, not seldom, men have vasectomies virtually on demand - one could also say of the Netherlands men aren't patronised much, unlike women - with minimal counselling. Paradoxically, it is quite possible that there will be more requests for reversal from the 43 men in our study who had a vasectomy because their partners from the ideal CS/TO group were denied the option of a TO (Table 3) than there would have been reversal requests from said partners if they had had a CS/TO.

If, in the above 2% reversal scenario for women on average 3 IVF cycles were to be applied (€3000 each, 3 cycles are covered by the basic health insurance), then the number of IVF cycles in the country (presently nearly 18,000 annually) would increase by 1.3%. This approach - easy well counselled access to opportunistic TO and easy access to IVF - would prevent annually at least 300–400 future unintended pregnancies and the costs and side-effects involved in the contraception for 4000 women. Said 2% serious regret scenario would cost €18,000 for 6 IVF cycles per 100 women who had CS/TO. Without access to opportunistic TO on the other hand, if these 100 women would instead have all hysteroscopic TOs sometime after delivery, this would cost currently (January 2011) €142,500 (plus €9000 for IVF cycles for the assumed associated serious regret rate of 1%) or €33,000 (plus ±€7000 reversal, sometimes ICSI if there were 2.4% regret [43]) if their partners would have vasectomies. If these 100 women would use instead LNG-IUDs for on average 18 years this would cost ±€115,000 and soon possibly, one fears, much more (see above). Copper IUDs would cost ±€46,000 over the same period, etonogestrel-releasing contraceptive implants (5 year implant unavailable in the Netherlands)±€182,000, three-monthly injections ±€ 108,000 and generic, second generation contraceptive pills €41,500. The above assumes a 100% continuation rate for reversible methods and takes into consideration that there are for example 2 Copper IUDs or 3 LNG-IUDs or 5 implants required if a woman needs 13 years contraception. Therefore it seems that the modern alternative methods to CS/TO are two to eight times more expensive (if our serious regret estimate is correct), and more unreliable because much more mistake-prone, mainly but not exclusively because of initiation delay[21].

### How best to arrange for TO

We suggest, taking the many suggestions of our participants in consideration, that parous pregnant women who are – arbitrarily – ≥28 years, likely to have ≥2 viable children after delivery, and in a stable relationship should be routinely informed together with their partners in the form of a pamphlet of the possibility of having a TO in combination with a (always conceivable) CS. This includes women who have only delivered vaginally previously. It would be appropriate to check later, preferably in the second trimester (as most of our respondents suggested), whether the information was read and understood and to record intentions in the notes. Attention will also be needed for sub fertile couples who hope to complete their family with the current pregnancy because these couples may be inclined not to see much need for sustained contraception after delivery. Such couples are at extra risk of surprise pregnancies that could place them in a sad dilemma. While in this group the desire for another child will often anyway -TO or not - result in IVF/ICSI.

If CS/TO counselling happens routinely, women/couples are unlikely to take offence. This is suggested by the observation that 25 years ago while asking about smoking, alcohol use and HIV/syphilis screening in pregnancy this was considered offensive by some women. This is no longer an issue because such questions and procedures are now part of routine medical care and are therefore not taken personally. Recording in the medical notes that the CS/TO subject has been discussed also prevents needless (then not seldom irritating) discussion of the subject in subsequent meetings and misunderstandings at delivery time, although confirmation just before the CS seems good practice. It would be helpful if the antenatal forms, also those used by midwives (see Table 2), have a standard (around 55% of all Dutch deliveries involve the ≥2^nd^ child), pre-printed area that can be filled in by the first person to ask the woman about whether she has been informed of the CS/TO combination in case a CS might turn out to be necessary. Many respondents emphasised that tact is needed when broaching the issue of CS/TO.

### Technical aspects

Many obstetricians believe that periparum TOs have higher failure rates. This is unproven, the large prospective US CREST study found the opposite [31]. Even if there was a higher failure rate with CS/TO that would be overwhelmingly offset by the pregnancies occurring before an intended interval TO or other (on)reliable method is realised, if ever. There are indications however that it is better to use sutures than clips for a peripartum TO because of the increased diameter of the tubes [19]. The former option is also marked cheaper, especially compared to the use of disposable applicators.

### Strengths and limitations

Strengths of our study include the high response rate and the obvious enthusiasm of the respondents to help us resolve our research question as expressed in the introductory letter we sent them (see last sentence “Introduction”). Furthermore the research question is more or less unique. Not detecting the regret rate of women who had a TO, but the regret rate of having had an informed choice or not was our aim. The limitations of our study include whether our results are applicable to other (Western) countries or even urban areas of the Netherlands. Our participants were living in villages and small towns surrounding a district hospital and culturally quite similar. A comparable survey in the urbanised areas of the Netherlands would have the advantage to study a more diverse group of previous patients – possibly some more vulnerable to coercion or less able to organise themselves an IVF in unforeseen circumstances – but the results would be very likely much more difficult to interpret due to a much lower response rate, partly because women would be far less inclined to identify with a specific hospital and its obstetrical staff. City women might be much more suspicious when presented with a questionnaire and also when – if our recommendations were to be implemented – counselled about CS/TO, unless it was clearly a routine procedure pertaining to all Para ≥1. A shift to more reliable contraception, to be provided especially at opportune moments [21], seems however particularly important in the cities because there the, multicultural, residents have much higher induced abortion rates (some groups 7–8 times higher than the population living in the area we studied [17]) and are often less inclined to be satisfied with oral contraceptives, vasectomies or IUDs.

### In summary

The decision to deliver by a CS is often only made or confirmed close to birth. Many obstetricians in the Netherlands and elsewhere find it unethical to talk about the option of a peripartum TO at this stage. However, earlier counselling about this option generally does not occur either. It certainly is seldom part of the established antenatal routine where the midwife or doctor, prompted by a pre-printed area in the antenatal notes, combines information about the chance that pregnancies may end in a CS with counselling about the inherent CS/TO option. In our study, as a result, 78% (119/152) of the women who would have wanted a CS/TO combination missed the opportunity to have one, with the consequence that some later had unintended pregnancies and others remain at risk. Providing the CS/TO option is, we think we demonstrated, also the more economical approach. Our survey has shown that the reluctance to counsel candidate women/couples about the CS/TO combination seems not evidence-based. Indeed, it cannot be evidence-based because there is no objective way to compare the non-financial costs of unintended pregnancies to the burden of – considerably fewer – regretted sterilisations. However, our local findings make it likely that a policy of never giving parous women an informed choice about CS/TO results many times (even a factor 62 to186) more often in disappointment than always giving them in time that choice. Moreover, our respondents, who are because of their personal experiences arguably in the best position to offer an opinion on this issue, believed – while non-pregnant – that pregnant women with their partners are quite capable and should be allowed, to decide for themselves.

We therefore ask readers to consider the possibility that the available evidence at the moment does not justify denying women/couples, at least in the Netherlands, the chance to make an informed choice about CS/TO. The onus is on the doctors/midwives who are reluctant to counsel parous pregnant women/couples about the possibility of CS/TO to support their position by performing – ideally prospective – cohort studies that compare the regret rates of women who were or were not given the option of having a CS/TO, and also contrast the impact of unintended pregnancies with the impact of reversal procedures. In the absence of such supporting evidence, we advocate not to ignore the informed consent maxim and to utilise the findings of our study to provide the appropriate written information and to initiate a discussion in the second trimester of every relevant pregnancy about the conceivability of a CS and its inherent TO option.

## Supporting Information

Supporting Information S1 Letter of introduction, Dutch. The introduction letter as we attempted to send to all 515 potential participants, and the three questionnaires (in Dutch and translated in English) namely for women who were sterilised (S2–S3), for women who were not sterilised but whose index delivery was a CS (S4**–**S5), and for women who had an earlier CS (S6–S7) but whose index delivery was vaginal.
(0.04 MB DOC)

Supporting Information S2 Questionnaire bevalling met sterilisatie.
(0.04 MB DOC)

Supporting Information S3 Questionnaire CS with sterilisation.
(0.05 MB DOC)

Supporting Information S4 Questionnaire keizersnede zonder sterilisatie.
(0.04 MB DOC)

Supporting Information S5 Questionnaire CS without sterilisation.
(0.05 MB DOC)

Supporting Information S6 Questionnaire vaginale bevalling, eerder keizersnede.
(0.05 MB DOC)

Supporting Information S7 Questionnaire vaginal delivery, CS earlier.
(0.05 MB DOC)
