# Supplementary material for: The Right to Informed Choice. A Study and Opinion Poll of Women Who Were or Were Not Given the Option of a Sterilisation with Their Caesarean Section
Source: PLoS One. 2011 Mar 22;6(3):e14776. doi: 10.1371/journal.pone.0014776 (PMC3062542; doi:10.1371/journal.pone.0014776)
Supplement: Supporting Information S1 — Letter of introduction, Dutch. The introduction letter as we attempted to send to all 515 potential participants, and the three questionnaires (in Dutch and translated in English) namely for women who were sterilised (S2–S3), for women who were not sterilised but whose index delivery was a CS (S4–S5), and for women who had an earlier CS (S6–S7) but whose index delivery was vaginal. (0.04 MB DOC) [file pone.0014776.s001.doc]

**Hoogeveen, oktober 2006**

**Deelname onderzoek ‘keizersnede & sterilisatie ?”**

Zeer geachte mevrouw,

Als gynaecologen van ziekenhuis Bethesda willen wij graag een beter inzicht krijgen in hoe wij vrouwen - die mogelijk een keizersnede gaan doormaken – het beste kunnen voorlichten over anticonceptie-mogelijkheden voor de periode daarna.

We zoeken naar een manier om dit zo zorgvuldig mogelijk te doen. U kunt ons daarbij helpen!

U bent in het verleden bevallen via een keizersnede en daarom “ervaringsdeskundige”.

U zou ons daarom heel goed kunnen adviseren bij een lastig probleem.

**Als gynaecologen proberen we namelijk uit te zoeken of het verantwoord/gewenst is om met een zwangere vrouw, die (mogelijk weer) met een keizersnede gaat bevallen en al minstens 1 kind heeft en niet erg jong is, te bespreken of zij eventueel samen met die nieuwe keizersnede een sterilisatie wil.**

**Achtergrond**

Vrouwen/partners met een compleet gezin, moeten vaak nog jaren (in principe tot het vijftigste jaar van de vrouw) oppassen dat ze niet zwanger worden en dit gaat wel eens fout. Als het fout gaat zijn er soms moeilijke keuzes te maken. Een sterilisatie kan zo’n dilemma voorkomen.

Een sterilisatie is zoals u weet het afbinden van de eileiders. Het is heel eenvoudig om de eileiders te sluiten als de baarmoeder toch zichtbaar is tijdens een keizersnede.

Een sterilisatie is ook de meest patiëntvriendelijke methode, je hoeft er meestal niet meer naar om te kijken. De hormonen en dus de menstruaties blijven onveranderd. Op wereldniveau is sterilisatie de meest voorkomende methode van anticonceptie. In landen als Amerika, Canada en Engeland wordt de keuzemogelijkheid van een sterilisatie tijdens een keizersnede bijna altijd besproken, als er al minstens 1 kind is. In ons land gebeurt dat veel minder.

Het kan natuurlijk zijn dat vrouwen meer geneigd zijn impulsieve beslissingen te nemen als zij zwanger zijn. Verder is het natuurlijk nooit 100% zeker dat de baby het eerste jaar zal overleven. Daarnaast kan de relatie ook mislopen. Misschien krijgen vrouwen dan spijt en hebben zij dan weer een operatie nodig om de eileiders te openen; een operatie die maar 70% succes heeft en een operatie is nooit leuk. Als een vrouw bij de 30% hoort waarbij het terugdraaien geen succes heeft, dan moet zij – indien ze nog steeds zwanger wil worden - de last van een reageerbuis-bevruchting doormaken.

**Wel of niet bespreken?**

Het gevolg van dit alles is, dat de keuzemogelijkheid van een sterilisatie bij een keizersnede voor het 2e, 3e, 4e, etc. kind in sommige Nederlandse ziekenhuizen bijna altijd en in andere bijna nooit met de patiënte besproken wordt. Niemand weet wat nu eigenlijk de juiste benadering is.

Een typische situatie is die waarin een vrouw tegen het einde van de 2e of 3e zwangerschap een dwarsligging blijkt te hebben. Zij en haar partner worden door de gynaecoloog geïnformeerd dat een bevalling met een keizersnede noodzakelijk is (vaak nadat een poging om de baby te draaien mislukt is). Moet de gynaecoloog hen voorlichten over de mogelijkheid om tijdens de keizersnede een sterilisatie te laten doen? Als zij/hij dat doet, liefst in aanwezigheid van haar partner, dan zullen haar leeftijd, de stabiliteit van haar relatie, de bedenktijd, het aantal kinderen en de kans dat de komende baby er gezond uit zal komen, natuurlijk belangrijke factoren zijn.

Er is bij een keizersnede altijd een kinderarts aanwezig die het kind snel onderzoekt om te zien of het op het eerste gezicht gezond is of niet, voordat eventueel een sterilisatie plaats vindt.

Een andere situatie is die waarin een vrouw die al eens per keizersnede beviel, nu gaat proberen om normaal te bevallen. Zij heeft ongeveer 70% kans dat dat lukt. Maar zou het een goed idee zijn, om vooraf te bespreken of zij ook een sterilisatie zou willen, als gewoon bevallen niet blijkt te lukken en er weer een keizersnede nodig is?

In zo’n geval is het mogelijk om dit onderwerp al heel vroeg in de zwangerschap aan te snijden.

**Onderzoek**

Is het verantwoord om het onderwerp sterilisatie überhaupt tijdens de zwangerschap aan te snijden? Dat is de vraag die wij proberen met uw hulp te beantwoorden. Wij sturen daarom bijgaande vragenlijst aan al onze patiënten, die een keizersnede hebben gehad, al dan niet in combinatie met een sterilisatie. Een van ons heeft al eens in een ander land onderzoek gedaan bij bijna 800 patiënten met verscheidene kinderen. Dit om te kijken of patiënten die wel of niet gevraagd waren of ze een sterilisatie wilden tijdens een keizersnede, tevreden waren. Deze vraag moet ook voor Nederland beantwoord worden, vinden wij.

**Onze vraag aan u is dus niet:**

**moeten vrouwen in dat soort omstandigheden gesteriliseerd worden?**

**Maar onze vraag is:**

**moet sterilisatie besproken worden als een mogelijkheid, waarvoor gekozen kan worden, mits er een redelijke bedenktijd is**?

U hebt ervaring met een keizersnede met of zonder sterilisatie en u kunt daarom ons en andere gynaecologen helpen om het juiste beleid te bepalen. Als u wat van uw tijd wilt besteden aan het zo zorgvuldig mogelijk invullen van bijgevoegde vragenlijst (zo mogelijk samen met uw partner), dan helpt u ons een verantwoord advies hierover te geven en zijn wij u zeer dankbaar.

Als een groot deel van de meer dan 500 vrouwen die we net zoals u aangeschreven hebben, met deze studie meewerkt, dan zal het resultaat gepubliceerd worden in een blad zoals het Nederlands Tijdschrift van Geneeskunde. Uw mening heeft dan invloed op de houding van de Nederlandse artsen/gynaecologen/verloskundigen, ten opzichte van vrouwen die mogelijk een keizersnede nodig hebben. Hoe meer de aangeschreven vrouwen meewerken, hoe betrouwbaarder we de visie van vrouwen, die al eens een keizersnede doormaakten, kunnen weergeven.

Nogmaals: er komt geen naam van de deelnemende vrouwen in en zij zijn ook niet op een andere manier te herkennen. Dit onderzoek is besproken en goedgekeurd door de Raad van Bestuur van het ziekenhuis en de medisch-ethische commissie.

**Als u niet mee wilt doen met dit (denken wij) belangrijke onderzoek, wilt u de vragenlijst dan oningevuld terugsturen? Dan vallen wij u ook niet lastig met herinneringsbrieven.**

Als bijlage treft u een gefrankeerde en geadresseerde retourenvelop aan waarin de vragenlijst zit. Die kunt u zo eenvoudig terugsturen: ingevuld als u mee wilt werken, niet ingevuld als u niet wilt meewerken.

Hebt u nog vragen bel dan de secretaresse van de gynaecologen 0528286222 of e-mail naar [verkuyl.d@bethesda.nl](mailto:verkuyl.d@bethesda.nl)

Mocht u geïnteresseerd zijn in het artikel, dat wij bij voldoende deelname kunnen publiceren, vul dan uw (e-mail) adres in, dan sturen we het u te zijner tijd toe.

Gynaecologen ziekenhuis Bethesda
