# Supplementary material for: The Right to Informed Choice. A Study and Opinion Poll of Women Who Were or Were Not Given the Option of a Sterilisation with Their Caesarean Section
Source: PLoS One. 2011 Mar 22;6(3):e14776. doi: 10.1371/journal.pone.0014776 (PMC3062542; doi:10.1371/journal.pone.0014776)
Supplement: Supporting Information S2 — Questionnaire bevalling met sterilisatie. (0.04 MB DOC) [file pone.0014776.s002.doc]

**VRAGENLIJST**

**Betreft bevalling dd. - - met sterilisatie**

*Wilt u het juiste antwoord* *omcirkelen en/of invullen?*

1. Hoeveel kinderen heeft u nu in totaal? **Jongens....... Meisjes.......**
2. Zijn al uw kinderen van dezelfde partner? **Ja / Nee**
3. Hebt u nog dezelfde partner als toen met de bovenstaande bevalling?

**Indien nee** : Kinderen .... van de eerste en .... van de tweede partner

1. Uw laatste bevalling is geëindigd in een keizersnede met een sterilisatie.

Vindt u het achteraf een verstandig besluit om tegelijkertijd een sterilisatie te krijgen? **Ja / Nee** (als Nee ga naar **II**)

**I. als** **Ja**:

Wat typeert uw situatie/mening? *(zo nodig meer dan één antwoord omcirkelen)*

**A** Ik wil geen kinderen meer en ik vind dat gedoe om met een andere methode een zwangerschap te voorkomen maar lastig

**B** Wij zouden weinig moeite hebben gehad om een andere betrouwbare methode te gebruiken maar dit was een handige gelegenheid

**C** Ik ben bang dat ik nog zwanger word omdat zo’n sterilisatie niet 100%

betrouwbaar is.

**D** Ik ben nu niet bang dat ik nog zwanger word, een sterilisatie is een stuk zekerder dan de methoden die ik vroeger gebruikte

**E** Ik vind het heel verstandig van ons dat mijn partner en ik zelf het initiatief

genomen hebben om een sterilisatie te vragen voor bij de keizersnede

**F** Ik vroeg om een sterilisatie maar de gynaecoloog wilde eerst niet / raadde het af

**G** De gynaecoloog sneed het onderwerp sterilisatie aan, maar deed ook zijn best het direct uit mijn hoofd te praten.

**H** De gynaecoloog sneed het onderwerp sterilisatie aan en liet ons zonder druk zelf beslissen

**I** Als ik 5 jaar jonger was geweest tijdens de laatste bevalling had ik geen

sterilisatie gewild

**J** Er was een goede medische reden voor de sterilisatie

**K** Ik ben toen de baby jong was bang geweest dat mijn baby iets zou overkomen en dat ik dan geen nieuw kind meer kon krijgen.

**L** De gynaecoloog sneed het onderwerp sterilisatie aan en probeerde ons te overtuigen om met een sterilisatie in te stemmen.

**M Anders**, nl:.................................................................................... .................................................................................................

**II**. Als u spijt heeft van de sterilisatie bij uw keizersnede

*(Graag alle antwoorden die op u van toepassing zijn, omcirkelen*)

**A** Ik wil nog meer kinderen

**B** Ik wil de mogelijkheid hebben nog meer kinderen te krijgen

**C** Ik wil niet meer kinderen maar vind het idee dat ik ze niet meer kan krijgen

vervelend

**D** Het was meer mijn partner’s beurt om iets te laten doen. **E** Ik heb een nieuwe relatie. **F** De vraag of ik een sterilisatie wilde heeft mij overrompeld en ik heb toen te

te haastig ja gezegd

**G** Ik was te jong

**H** Ik heb lichamelijke klachten waarvan ik de sterilisatie de schuld geef

**I** Ik heb psychische klachten waarvan ik de sterilisatie de schuld geef

**J** Soms heb ik wel eens even spijt maar alles bij alles genomen was het toch een goede beslissing

**K** Ik heb spijt omdat ik niet beiden, dus een jongetje **en** een meisje heb

**L** Er was een goede medische reden voor de sterilisatie

**M** Ik ben onder druk gezet om gesteriliseerd te worden

**N** Andere reden, nl: ..................... ..................................................... . ................................................................

1. Een sterilisatie tijdens een keizersnede is eenvoudig. Vindt u dat deze mogelijkheid besproken moet worden met de zwangere en haar partner:

**ja,** want ................................... ........................................................................... ...................................................

**nee**, want...................................................................................................... ..........................................................

6. Als u de vorige vraag met **ja** beantwoord heeft, vindt u dan dat bespreken voor het eerst gebeuren moet met de keizersnede voor de 2^e^, 3^e^, 4^e^, 5^e^, 6^e^, 7^e^, 8^e^, 9^e^ of 10 baby ? *(Graag juiste aantal omcirkelen*)

7. Vindt u dat de gemiddelde Nederlandse vrouw, samen met haar partner, in de laatste dagen van de zwangerschap, in staat is om een verantwoorde beslissing te nemen over wel of geen sterilisatie tijdens een keizersnede?  **Ja** / **Nee** (Maar……………………………………………………….)

8. Vindt u dat de verloskundige, gynaecoloog of huisarts er goed aan doet de mogelijkheid van een sterilisatie **in het begin** van de zwangerschap bij vrouwen die al kinderen hebben aan te kaarten? *(Zo van:“stel dat u (weer) een keizersnede nodig blijkt te hebben en een stevige baby komt er gezond uit, zou u er vast over willen denken of u dan eventueel een sterilisatie zou willen?“)*

Goed om die vraag te stellen: **Ja** / **Nee**

9 **A.** Door wie werd bij uw laatste zwangerschap de vraag voor het eerst gesteld of u ook een sterilisatie wilde?

Door : **verloskundige / huisarts / gynaecoloog / uzelf**

*(omcirkel juiste antwoord)*

Wanneer was dat? *(omcirkel juiste antwoord)*

**a.** Voor de zwangerschap

**b.** Vroeg in de zwangerschap

**c.** Midden zwangerschap

**d.** Laatste weken

**e.** Laatste dagen

**f.** Laatste uren

Hoe heeft u de timing ervaren?......... Was dit het juiste moment?...........

9 **B.a.** Zijn er mensen in uw omgeving die vinden dat u dom was om u te laten

steriliseren? **Ja / Nee**

**b.** Zijn er mensen in uw omgeving die juist vinden dat u die sterilisatie goed

geregeld heeft ? **Ja / Nee**

1. Toen u de optie van een sterilisatie aangeboden heeft gekregen was dat een min of meer neutraal aanbod of werd er druk op u uitgeoefend om gesteriliseerd te worden?

**Neutraal aanbod / druk om niet / druk om wel gesteriliseerd te worden/**  **was mijn eigen idee**

Licht eventueel toe .................................................................................

**D.** Is er druk door uw **omgeving** uitgeoefend om een sterilisatie te krijgen? **Ja / Nee**

**E**. Is er druk door uw **omgeving** uitgeoefend om **geen** sterilisatie te krijgen?**Ja / Nee**

10. Bent u ooit per vergissing zwanger geworden ? **Ja / Nee**

Welk **Jaar**? ……..

11.Ik vind het niet tot de taak van de dokter behoren het onderwerp anticonceptie

aan te snijden, als ik iets wil, of wil weten neem ik **zelf** het initiatief wel. **Ja / Nee**

12. a.**1** Wat voor **methoden** gebruikte u vroeger om niet zwanger te worden?

(**omcirkel** alles ooit gebruikt plus **schrijf** achter methode hoelang)

pil, spiraaltje, zingen en kerk etc.

injectie, kalender methode, inplant,

condoom, man gesteriliseerd, anders, nl:........................,

alleen borstvoeding, onthouding

**2.** Ik en/of mijn partner maakte nogal eens fouten met de methode die we

vroeger gebruikten. **Ja / Nee**

b. Had u klachten over de methoden die u vroeger gebruikte? **Ja / Nee**

Van wat………………………….. welke …………………….. klachten?

Van wat………………………….. welke …………………….. klachten?

Van wat………………………….. welke …………………….. klachten?

Van wat………………………….. welke …………………….. klachten?

13. Heeft u klachten over de sterilisatie? **Ja / Nee**

Zo ja, **welke:** ................................................................................................................

......................................................................................................................................

14. Neem het voorbeeld uit de begeleidende brief van de zwangere dame met 2 kinderen wier derde baby dwars ligt.

Er is geen haast en de gynaecoloog bespreekt de mogelijkheid van een sterilisatie met de komende keizersnede **niet**.

**Vindt u dat**: verstandig / een misser / bevoogdend? (**omcirkel** juiste graag)

15. Neem het voorbeeld uit de begeleidende brief van de zwangere dame met 2

kinderen wier derde baby dwars ligt.

Er is geen haast en de gynaecoloog bespreekt de mogelijkheid van een sterilisatie met de komende keizersnede **wel**

**Vindt u dat**: verstandig / een misser / bevoogdend/ bemoeizuchtig?  *(omcirkel juiste antwoord)*

16. Vindt u in het algemeen dat bij een compleet gezin en een man die 2 jaar ouder is, dat bij een sterilisatie beter **de man / de vrouw** gesteriliseerd kan worden?

17. **Hebt** u nog opmerkingen/suggesties/klachten ? .......................................................................................................................... ............................................................................................................................

**Heel hartelijk bedankt voor uw medewerking**

Vakgroep gynaecologen Hoogeveen

(Als u een verslag van de studie wilt, dan hier graag **uw e-mail adres** vermelden: ………………..@........................
