# Supplementary material for: The Right to Informed Choice. A Study and Opinion Poll of Women Who Were or Were Not Given the Option of a Sterilisation with Their Caesarean Section
Source: PLoS One. 2011 Mar 22;6(3):e14776. doi: 10.1371/journal.pone.0014776 (PMC3062542; doi:10.1371/journal.pone.0014776)
Supplement: Supporting Information S3 — Questionnaire CS with sterilisation. (0.05 MB DOC) [file pone.0014776.s003.doc]

**Questionnaire for women who had a delivery with sterilisation**

Delivery on   - -

**Please circle or fill in the appropriate answer(s).**

1. How many living children do you have in total? ….**Boys**, …… **Girls.**
2. Are all your children from the same partner? **Yes/No**

If not: ……. from the first and ……. from the second partner.

3. Do you still have the same partner as you did at the above delivery? **Yes/No**

4. Your last delivery resulted in a CS with a sterilisation. In retrospect, do you think this     sterilisation was a good decision? **Yes / No** (if you answered **No**, go straight to **II**)

**I. If you answered Yes:** What characterises your situation/opinion:
 (You may circle more than one answer if necessary.)

**A.** I do not want to have more children and I felt burdened by having to arrange        contraception.

**B.** My partner and I would have had little trouble using another reliable contraception method,          but this was a convenient opportunity.
**C.** I worry that might still become pregnant because a sterilisation is not 100% reliable.
**D.** I am not worried that I will become pregnant now because a sterilisation is much more     reliable than the methods that I used to use.
**E.** I think it was very sensible of me and my partner to take the initiative in asking for a        sterilisation with my CS.  **F.** I asked for a sterilisation but the obstetrician did not want to do it at first/tried to dissuade me.
**G.** The obstetrician raised the subject of having a sterilisation with the CS but tried to dissuade     me at the same time. **H.** The obstetrician raised the subject of having a sterilisation with the CS and let us decide by     ourselves and without pressure.
**I.** If I had been 5 years younger at my last delivery, I would not have wanted a sterilisation.
**J.** There was a good medical reason for a sterilisation.
**K.** When my baby was young I was sometimes scared that something would happen to it and      that I wouldn’t be able to get another child.
**L.**  The obstetrician raised the subject of having a sterilisation with the CS and tried to convince     us to agree to a sterilisation.
**M.** Other: …………………………………………………………………..………………………
 ……………………………………………………………………….……………………………..

**II**. If you regret having had a sterilisation with your CS, please circle **all** the answers that characterise your situation/opinion:

**A.**  I want more childrenn How many? ………

**B.**  I want the option of having more children.

**C.**  I don’t want more children, but do not like the idea of not being able to have more.

**D.** It was actually my partner’s turn to have something done.

**E.**  I have a new partner.

**F.** The question of whether I wanted a sterilisation took me by surprise and I said yes too hastily.

**G.**  I was too young.
**H.** I have physical problems and I blame my sterilisation.
**I.**  I have psychological problems and I blame my sterilisation.

**J.** Sometimes I do regret my sterilisation a little bit, but all in all it was a good decision.
**K.**  I regret the sterilisation because I do not have both a girl and a boy.
**L.** I regret the sterilisation although there was actually a good medical reason for it.
**M.**  I was pressured into having a sterilisation.
**N.**  Other: ...........................................................................................................................................

..........................................................................................................................................................

5. Performing a TO during a CS is easy. Do you think this option should be discussed with a pregnant woman and her partner?: **Yes /No**

**Because** ...............................................................................................................................................
…………………………………………………………………………………………………………………………….

6. If you answered the previous question with **Yes**, do you think this should be discussed for the first time before the CS for the 2nd, 3rd, 4th, 5th, 6th, 7th, or 8th child?
(Circle the number you prefer)

7. Do you think that the average Dutch woman is able, together with her partner, **in the last days of her pregnancy**, to make a responsible decision about whether to have a sterilisation combined with her CS?  **Yes**/**No,** but **(optional)**……………………………………………………………..
……………………………………………………………………………………………………..

8. Are you of the opinion that a midwife, obstetrician or GP should discuss **early during pregnancy** the option of sterilisation with women who already have children? 
(Something like: *“Suppose you happen to need a CS (again) and a healthy, strong baby is delivered, could you please consider in the months to come whether you would also like a sterilisation?”*)

Is such a question appropriate? **Yes**/**No**

9. **A.** During your last pregnancy, who asked you whether you also wanted to be sterilised?

**The midwife/ general practitioner/gynaecologist/I raised the subject myself.**  (Please choose one of these four options)

When was that?

**a.** Before the pregnancy

**b.** Early on in the pregnancy

**c.** Mid-pregnancy **d.** In the last weeks

**e.**  In the last day’s **f.** In the last hours

How did you feel about this timing? …………….…………………………………………………..

Was it the right moment?.....................................................................................................................

**B.** 1. Are there people in your environment who think you made a bad decision with regard to   getting a sterilisation? **Yes/No**

2. Are there people in your environment who think you were clever in having a sterilisation   this way? **Yes/No**

**C.** When you were offered the option of sterilisation: was this a more or less neutral offer, or did you feel pressured to make a certain decision?

**Neutral offer/ pressure to get sterilised/pressure to not get sterilised/it was my own idea**

Room to elaborate (optional) .............................................................................................................
............................................................................................................................................................

**D.** Did your environment make you feel pressured to get a sterilisation? **Yes/No**

**E.** Did your environment make you feel pressured to **not** get a sterilisation? **Yes/No**

10. Have you ever become pregnant by mistake? **Yes/No**

In what year? ……..

11. I am of the opinion that a doctor should not raise the subject of contraception. If I want something or want to know something, I will take the initiative myself: **Yes/No.**

12. a.**1** In the past, what method(s) have you used to prevent pregnancy?
 (Circle all the methods you ever used and write down for how long you used each method)

contraceptive pill intra uterine device (coil), implant contraceptive injection fertility awareness (calendar) withdrawal

condom partner had a vasectomy other…………..…..

just lactation abstinence

**2.** Me and/or my partner quite often made mistakes with the method that we used in the past**. Yes/No**

.

b. Did you have problems with any of the methods that you used in the past? **Yes/No**

Method………………………… problem(s) ……………………………………………
 Method………………………… problem(s) ……………………………………………

Method………………………… problem(s) ……………………………………………

Method………………………… problem(s) …………………………………………….

13. Do you have any complaints about your sterilisation? **Yes/No**

If so, what complaints?.........................................................................................................

...............................................................................................................................................
 ..............................................................................................................................................

**In the questions below, please circle the right answer.**

14. Consider the example of the enclosed letter involving a woman with 2 children whose third is lying in a transverse position. There is no hurry and the obstetrician does not counsel her about the option of a sterilisation with the coming CS. 

Do you find that**: sensible/a mistake/patronising**

15. Consider the example from the enclosed letter involving a woman with 2 children whose third is lying in a transverse position. There is no hurry and the obstetrician does counsel her about the option of a sterilisation with the coming CS.

Do you find that**: sensible/a mistake/patronising/meddlesome**

16. Generally speaking, do you think that in a complete family, with a man who is two years older than the woman, it is better that **the man** or **the woman** gets sterilised?

17. Do you have other remarks/suggestions/complaints? ............................................................................................................................................ ..............................................................................................................................................

..............................................................................................................................................

..............................................................................................................................................

**Thank you very much for your cooperation**

Obstetricians, Hoogeveen

Optional : write down your **e-mail address** if you want a summary of the results of this study:

…………………………@………………………………
