# Supplementary material for: The Right to Informed Choice. A Study and Opinion Poll of Women Who Were or Were Not Given the Option of a Sterilisation with Their Caesarean Section
Source: PLoS One. 2011 Mar 22;6(3):e14776. doi: 10.1371/journal.pone.0014776 (PMC3062542; doi:10.1371/journal.pone.0014776)
Supplement: Supporting Information S4 — Questionnaire keizersnede zonder sterilisatie. (0.04 MB DOC) [file pone.0014776.s004.doc]

**Vragenlijst : Vrouwen die een keizersnede hadden (u op --- --- --- )**

**zonder sterilisatie**

Gaarne, het/de juiste antwoord**(en**) omcirkelen en invullen.

1. Hoeveel kinderen heeft u nu in het **totaal?** .....          **Jongens**.......    **Meisjes**.......

2. Zijn al uw kinderen van dezelfde partner?   **Ja/ Nee**

                                  Als nee :          ....  van de **eerste** en.... van de **tweede partner**

3. Hebt u nog dezelfde partner als toen u beviel van uw laatste baby?   **Ja / Nee**

4. Uw laatste bevalling is geëindigd in een keizersnede had u die met een sterilisatie

    gecombineerd gehad willen hebben?   **Ja**  /  **Nee**   (als **Nee** ga naar **II**)

**I.   Als Ja:**

                    Wat typeert uw situatie: (zo nodig **meer dan één antwoord** omcirkelen)

**A**.  Ik wil geen kinderen meer en ik vind dat gedoe om een zwangerschap te

      voorkomen maar lastig

**B**.  Het zou handig geweest zijn om een sterilisatie te combineren met de

      keizersnede maar mijn partner en ik hebben weinig moeite een betrouwbare

      methode te gebruiken.

**C**.  Ik ben bang dat ik nog per vergissing zwanger word.

**D**.  Ik zou nu wel een sterilisatie willen maar ik ben wel wat bang voor een operatie

**E**.  Ik zou nu wel een sterilisatie willen maar het komt er steeds niet van het te

      organiseren.

**F**. Een sterilisatie is te duur.

**G**. Ik vind het dom dat mijn partner en ik niet zelf het initiatief genomen hebben om

      een sterilisatie te vragen tegelijkertijd met de keizersnede, een gemiste kans.

**H**.  Ik vroeg om een sterilisatie maar de gynaecoloog wilde niet/raadde het af

**I**.   Nu heb ik spijt dat ik niet gesteriliseerd ben met de keizersnede, maar toen was

ik  bang dat mijn baby iets zou overkomen, dus wilde ik geen drastische  besluiten nemen

**J**.  Er was eigenlijk een goede medische reden voor een sterilisatie

**K**. De gynaecoloog sneed het onderwerp sterilisatie aan maar deed ook zijn best het

     direct uit mijn hoofd te praten, dat liet mij er helaas van afzien

**L**.  Anders, nl: ....................................................................................                                        ..........................................................................................................

**II**. Als u ***geen*** sterilisatie had gewild met uw keizersnede

   (Graag ***alle*** antwoorden  invullen die op u van toepassing zijn)

**A**.  Ik wil nog meer kinderen

**B**.  Ik wil de mogelijkheid openhouden nog meer kinderen te krijgen

**C**.  Ik wil niet meer kinderen maar zou het idee dat ik ze niet meer kan krijgen

      vervelend vinden

**D.**  Het was mijn partner’s beurt om iets te laten doen

**E**.  Ik heb weinig moeite een andere betrouwbare methode te gebruiken om niet

     zwanger te worden

**F**.  Mijn religie/cultuur staat een sterilisatie zonder goede medische reden niet toe

**G**. Als ik 5 jaar ouder was geweest tijdens de laatste bevalling had ik een sterilisatie

     gewild.

**H**.  De dokter heeft het niet gevraagd, ik had ook geen sterilisatie gewild maar ik vind

     dat hij/zij mij toch had moeten informeren over de mogelijkheid

**I.** Ook al had de dokter het mij gevraagd dan had ik zeker niet per vergissing ja

      gezegd

**J**.  Ik wacht tot ik minstens één jongen en één meisje heb voordat ik stop met

      kinderen krijgen

**K.**    De gynaecoloog sneed het onderwerp sterilisatie aan maar deed ook zijn best het direct uit mijn hoofd te praten, dat overtuigde mij

**L.**     Andere reden, nl: ..................... ............................................................................. .................................................

**III**  Kies alstublieft **één** van de onderstaande mogelijkheden die het best bij u past:

**a**.  De dokter vroeg mij **niet** of ik bij de keizersnede een sterilisatie zou willen en ik zou **nee** gezegd hebben denk ik. Ik zou daar nu **spijt** van hebben denk ik maar ik kon op dat moment niet alles overzien..

**b**.  De dokter vroeg mij **niet** of ik bij de keizersnede een sterilisatie zou willen en ik zou **nee** gezegd hebben denk ik. Ik zou daar nu **geen** spijt van hebben denk ik.

**c**.  De dokter vroeg mij **niet** of ik bij de keizersnede een sterilisatie zou willen en ik zou **ja** gezegd hebben denk ik. Ik zou daar nu **spijt** van hebben denk ik maar ik kon op dat moment niet alles overzien..

**d**.  De dokter vroeg mij **niet** of ik bij de keizersnede een sterilisatie zou willen en ik zou **ja** gezegd hebben denk ik. Ik zou daar nu **geen** spijt van hebben denk ik

**e**.  De dokter vroeg mij **wel** of ik bij de keizersnede een sterilisatie zou willen en ik heb **nee** gezegd. Ik heb daar nu **geen** spijt van.

**f**.  De dokter vroeg mij **wel** of ik bij de keizersnede een sterilisatie zou willen en ik heb **nee** gezegd. Ik heb daar nu **spijt** van maar ik kon op dat moment niet alles overzien..

**g**. Het was afgesproken dat ik ook een sterilisatie zou krijgen maar de dokter vergat het te doen. **Jammer**/ **gelukkig** maar (kies).

**h**. Ik vind het niet tot de taak van de dokter behoren het onderwerp van anti conceptie aan te snijden als ik iets wil of wil weten neem ik **zelf** het initiatief wel

**i**. Anders namelijk………………………………………………………………………………………….

5. Een sterilisatie tijdens een keizersnede is eenvoudig. Vindt u dat deze mogelijkheid  besproken moet worden met de zwangere en haar partner:

**ja,**  want ………………………………………………………………………. ………………………………………………………….............................................................................................

**nee**, want...................................................................................................................................................................

 ……………………………………………………..

6. Als u de vorige vraag met **ja** beantwoord heeft, vindt u dan dat bespreken voor het eerst  gebeuren moet met de keizersnede voor de: 1e 2e, 3e, 4e, 5e, 6e, 7e, 8e, 9^e^ of 10e baby                                                                                 (Graag juiste **omcirkelen**)

7.  Vindt u dat de gemiddelde Nederlandse vrouw, samen met haar partner, in de laatste dagen van de zwangerschap, in staat is om een verantwoorde beslissing te  nemen over wel of geen sterilisatie  tijdens een keizersnede?  

**Ja, want………………………………………………**

**Nee, want…………………………………………………**

8.  Vindt u dat de verloskundige, gynaecoloog of huisarts er goed aan doet de mogelijkheid  van een sterilisatie **in het begin** van de zwangerschap bij vrouwen die al kinderen hebben aan te kaarten? (Zo van: stel dat  u (weer) een keizersnede nodig blijkt te hebben en een stevige baby komt er gezond uit, zou u er vast over willen denken of u dan eventueel een sterilisatie zou willen?)                                     Goed om die vraag te stellen      **Ja** /  **Nee**

9  **A** Wanneer bij uw laatste zwangerschap werd bij u de vraag of u ook een sterilisatie wilde voor het eerst gesteld door uw **verloskundige /huisarts/ gynaecoloog/uzelf/ niemand**   (**omcirkel** juiste van deze vijf mogelijkheden)?

Was dat

**a**  Voor de zwangerschap

**b**  Vroeg in de zwangerschap

**c**. Midden zwangerschap                        **Omcirkel juiste**

**d**. Laatste weken

**e**. Laatste dagen

**f.**  Laatste uren

**g**. Nooit

Hoe heeft u de timing ervaren?......... ………………………… …………………………………………..

Was dit het juiste moment?.......... ……………………………………………

.

9 **B**  In geval u **niet** de optie van een sterilisatie aangeboden heeft gekregen, zou u j**a**  gezegd hebben als het u wèl gevraagd was?    **Ja / Nee /  Weet ik niet**

**Waarom**: ..............................................................................................................

**C**.  Als u **wel** de optie van een sterilisatie aangeboden heeft gekregen was dat een

       min of meer neutraal aanbod of werd er druk op u uitgeoefend ?  **Neutraal**

**aanbod / druk om niet / druk om wel gesteriliseerd te worden**

**Licht eventueel toe** .......................................................................................

**D**.   Is er druk door uw omgeving uitgeoefend om een sterilisatie te krijgen?  **Ja /**

**Nee**

**E**.  Is er druk door uw omgeving uitgeoefend om **geen** sterilisatie te krijgen?   **Ja /**

**Nee**

10.  Bent u ooit per vergissing zwanger geworden ? **Ja / Nee**    (Welk **Jaar**? ............)

11.Ik vind het niet tot de taak van de dokter behoren het onderwerp  anticonceptie aan te snijden. Als ik iets wil, of wil weten neem ik **zelf** het initiatief wel. **Ja / Nee**

12.   Wat voor **methode** gebruikt u tegenwoordig om niet zwanger te worden?

        (**omcirkel** aub)

**a**.**1,** pil,                               spiraaltje,                               geen seks    .

           injectie,                        kalender methode,                inplant

           condoom,                    man gesteriliseerd,                zingen en kerk enzo

           alleen  borstvoeding,   ik ben gesteriliseerd,            iets anders nl:....................

Als u later gesteriliseerd bent, wanneer was dat**?** 200?.  (**jaar** graag), Was dat samen met een bevalling? **Ja/Nee**

**2.** Ik/wij maken nogal eens fouten met de methode die we gebruiken   **Ja/Nee**

**b**. Geen **methode** want:

**1**. Ik denk dat ik/wij niet zwanger kan/kunnen worden,

**2**. Ik heb geen partner

**3**. Ik wil zwanger worden                                     (**omcirkel** juiste)

**4**. Ik neem risico?s

**5**. Ik zou het niet zo erg vinden als ik zwanger werd

**6**. Ik ben zwanger: **vergissing / geen vergissing**

**7**. Ik denk dat ik te oud ben om zwanger te worden

13.  Heeft u klachten over de methode die u gebruikt?     **Ja  / Nee**

           Zo ja, **welke:** ................................................................................................................

14.  Neem het voorbeeld uit de begeleidende brief van de zwangere dame met 2

 kinderen wier derde baby dwars ligt. Er is geen haast en de gynaecoloog bespreekt de mogelijkheid van een sterilisatie met de komende keizersnede **niet.**

**Vindt u dat**: verstandig / een misser / bevoogdend ? (**omcirkel** juiste graag)

15.  Neem  het voorbeeld uit de begeleidende brief  van de zwangere dame met 2 kinderen wier derde baby dwars ligt. Er is geen haast en de gynaecoloog bespreekt de mogelijkheid van een sterilisatie met de komende keizersnede **wel.**

**Vindt u dat**: verstandig / een misser / bevoogdend/ bemoeizuchtig?  (**omcirkel** juiste

                           graag)

16. Vindt u in het algemeen dat bij een compleet gezin en een man die 2 jaar ouder

      is, dat bij een sterilisatie beter **de man of de vrouw** gesteriliseerd kan worden?

17. Hebt u nog opmerkingen/suggesties/klachten (evnt. extra papier gebruiken?)

**Heel hartelijk bedankt voor uw medewerking**

Vakgroep gynaecologen Hoogeveen

**Vermeldt hier evt. uw e-mail adres** **:**    @

*(indien u graag een verslag van de uitkomst van deze studie wilt)*
