# Supplementary material for: The Right to Informed Choice. A Study and Opinion Poll of Women Who Were or Were Not Given the Option of a Sterilisation with Their Caesarean Section
Source: PLoS One. 2011 Mar 22;6(3):e14776. doi: 10.1371/journal.pone.0014776 (PMC3062542; doi:10.1371/journal.pone.0014776)
Supplement: Supporting Information S5 — Questionnaire CS without sterilisation. (0.05 MB DOC) [file pone.0014776.s005.doc]

**Questionnaire for women who have had a CS without sterilisation**

Delivery on   - -

**Please circle or fill in the appropriate answer(s).**

1. How many living children do you have in total? ….….**Boys,** ….….**Girls.**

2. Are all your children from the same partner? **Yes/No**

If not: ……. from the first and ……. from the second partner.

3. Do you still have the same partner as you did at the above delivery? **Yes/No**

4. Your last delivery ended in a Caesarean section (CS). Would you have wanted to combine it

with a sterilisation? **Yes**/**No** (If you answered **No,** go straight to **II**)

**I.** If you answered **Yes:** What characterises your situation/opinion
(You may circle more than one answer if necessary):

**A.**  I do not want to have more children and feel burdened by having to prevent falling pregnant.

**B.** It would have been convenient to have a sterilisation with my CS, but my partner and I have little        trouble using reliable contraception.
**C.** I worry that I will become pregnant unintendedly one of these days.
**D.** I would like a sterilisation now but am afraid of the operation.
**E.** I would like a sterilisation now but procrastinate organising it.
**F.** A sterilisation I could organise now is too expensive for me.
**G.** I think my partner and I lacked foresight when we didn’t take the initiative to get a sterilisation       with the CS. It was a missed opportunity. **H.** I asked for a sterilisation but the obstetrician advised against it/refused.
**I.**  I now regret that I did not have a TO at my CS, but at the time of delivery I was afraid something       could happen to my baby and did not want to make an irreversible decision.
**J.** There was actually a good medical reason for a sterilisation.
**K.** The obstetrician raised the subject of having a sterilisation with the CS but dissuaded me at the        same time. This, regrettably, decided me against having a sterilisation.
**L.** Other: ……………………………………………………………………..……………………….
 ……………………………………………………………………….………………………………..

**II.** If you had **not** wanted a sterilisation with your CS, please circle **all** the answers that characterise your situation/opinion:

**A.** I want more children. How many? ………

**B.**  I want the option of having more children.

**C.** I don’t want more children, but do not like the idea of not being able to have more.

**D.** It was my partner’s turn to have something done.

**E**. I have little trouble using a reliable method to prevent a pregnancy.

**F.** My religion/culture does not allow sterilisation without a good medical reason.

**G.** If I had been 5 years older with this CS, I would have liked a sterilisation. **H.** The doctor did not ask me. I would not have wanted a sterilisation but I think that he/she should        have asked me anyway.

**I.**  Even if the doctor had asked me I would definitely not have said yes in the confusion.

**J.** I am waiting until I have at least one boy and one girl before I stop having children.

**K.** The obstetrician raised the subject of having a TO with the CS but dissuaded me at the       same time. This convinced me not to have a sterilisation.

**L.** Other: ……………………………..……………………………….……………………... ……………………………………………………………………….………………………..

**III**. Please choose **one** of the following options that best applies to your situation (options continue on the following page):

**A.** The doctor did **not** ask me whether I wanted a sterilisation with my CS. I think I would have said **no.** I think I would **regret** that decision now, but I couldn’t oversee everything at the time.

**B.** The doctor did **not** ask me whether I would want a sterilisation if I had a CS. I think I would have said **no. I** think I would **not regret** that decision now.

**C.** The doctor did **not** ask me whether I would want a sterilisation if I had a CS. I think I would have said **yes.** I think I would **regret** that decision now, but I couldn’t oversee everything at the time.

**D.** The doctor did **not** ask me whether I would want a sterilisation if I had a CS. I think I would have said **yes.** I think I **would not regret** that decision now.

**E.** The doctor **did** ask me whether I would want a sterilisation with the CS. I said **no.** I do **not** **regret** that decision now.

**F.** The doctor **did** ask me whether I would want a sterilisation with the CS. I said **no**. I regret that decision now, but I could not oversee everything at the time.

**G.** It had been agreed that I would be sterilised if I had a CS but **fortunately/unfortunately** my doctor forgot to do it. (Choose one)

**H.** Other: ……………………………………………………………………………………………
……………………………………………………………………………………………………….

5. Performing a sterilisation during a CS is easy. Do you think this option should be discussed with a pregnant woman and her partner? **Yes/No**

**Because**……………………………………………………………………………………………………………………………………………………………………………………………………………..

6. If you answered the previous question with Yes, do you think this should be discussed for the first time before the CS for the 2nd, 3rd, 4th, 5th, 6th, 7th, or 8th child?
(Circle the number you prefer)

7. Do you think that the average Dutch woman is able, together with her partner, **in the last days of her pregnancy**, to make a responsible decision about whether to have a sterilisation combined with her CS? **Yes**/**No,** but **(optional)** ……………………………………………………………..……….
………………………………………………………………………………………………………...

8. Are you of the opinion that a midwife, obstetrician or GP should discuss **early during pregnancy** the option of sterilisation with women who already have children? (Something like: *“Suppose you happen to need a CS (again) and a healthy, strong baby is delivered. Could you please consider in the months to come whether you would also like a sterilisation?”*)
Is such a question appropriate? **Yes**/**No**

9. **A.** In what phase of your last pregnancy did your **midwife/GP/gynaecologist/yourself/nobody did** first ask whether you also wanted to be sterilised? (Please circle one of these five options)

Was that:

**a.** Before the pregnancy

**b.** Early on in the pregnancy

**c**. Mid-pregnancy

**d**. In the last weeks

**e**. In the last days

**f**. In the last hours

**g.** Never

How did you feel about this? ……………………………………………………………………………..

………………………………………………………………………………….…………………………

Was it the right moment? ………………………………………………………………...........................

…………………………………………………………………………………………………………….

1. If you **were not** offered the option of a sterilisation:

Would you have said yes if you had been offered one? **Yes/No/Don’t know**

**Why**:.................................................................................................................................................................................................................................................................................................................................

1. If you **were** offered the option of sterilisation:

Was this a more or less neutral offer, or did you feel pressured to make a certain decision?

**Neutral offer/pressure to get sterilised/pressure to not get sterilised**

Room to elaborate (optional) .............................................................................................................. ...............................................................................................................................................................

1. Did your environment make you feel pressured to get a sterilisation? **Yes**/**No**
2. Did your environment make you feel pressured to **not** get a sterilisation? **Yes**/**No**

10. Have you ever become pregnant unintendedly? **Yes/No**

In what year? ……..

11.    I am of the opinion that a doctor should not raise the subject of contraception. If I want something or want to know something, I will take the initiative myself: **Yes/No.**

12. **a**.**1.** What method are you presently using to prevent pregnancy? (Please circle)

contraceptive pill intra uterine device (coil) abstinence contraceptive injection fertility awareness (calendar) implant

condom partner had a vasectomy withdrawal

just lactation I have since had a sterilisation other:..................

If you were sterilised at a later stage in another hospital, when was that? 200… (**year**).
Was that in combination with a delivery? **Yes/No**

**2.** Me and/or my partner quite often make mistakes with the method that we are using. **Yes/No**

**b**. I don’t use **any** method because:

**1**. I don’t think that I/we can become pregnant

**2**. I don’t have a partner

**3**. I want to become pregnant **(circle the appropriate answer(s)) 4.** I take risks

**5**. I wouldn’t really mind becoming pregnant

**6**. I am pregnant (**a mistake/not a mistake)**

**7**. I think I am becoming too old to become pregnant

13. Do you have any complaints about the method you use? **Yes/No**

If yes, what complaints? ............................................................................................................................

..........................................................................................................................................................................................................................................................................................................................................

**In the questions below, please circle the answers that best reflect your opinion.**

14. Consider the example of the enclosed letter involving a woman with 2 children whose third is lying in a transverse position. There is no hurry and the obstetrician **does not** counsel her about the option of a sterilisation with the coming CS. 

Do you find that: **sensible/a mistake/patronising**

15. Consider the example from the enclosed letter involving a woman with 2 children whose third is lying in a transverse position. There is no hurry and the obstetrician **does** counsel her about the option of a sterilisation with the coming CS.

Do you find that: **sensible/a mistake/patronising/meddlesome**

16. In general, do you think that in a complete family, with a man who is two years older than the woman, it is better that **the man** or **the woman** gets sterilised?

17. Do you have other remarks/suggestions/complaints? ..........................................................................................................................................................................................................................................................................................................................................

**Thank you very much for your cooperation**

Obstetricians, Hoogeveen

Optional: write down your e-mail address if you want to see the results of this study:

…………………………@………………………………
