# Supplementary material for: The Right to Informed Choice. A Study and Opinion Poll of Women Who Were or Were Not Given the Option of a Sterilisation with Their Caesarean Section
Source: PLoS One. 2011 Mar 22;6(3):e14776. doi: 10.1371/journal.pone.0014776 (PMC3062542; doi:10.1371/journal.pone.0014776)
Supplement: Supporting Information S6 — Questionnaire vaginale bevalling, eerder keizersnede. (0.05 MB DOC) [file pone.0014776.s006.doc]

*Vrouwen die eerder een keizersnede hadden. Betreft vaginale bevalling van - -*

Gaarne, als u mee wilt doen, het/de juiste antwoord**(en**) omcirkelen en invullen.

1. Hoeveel levende kinderen heeft u nu in het **totaal? ... , Jongens.... Meisjes**....

2. Zijn al uw kinderen van dezelfde partner? **Ja/ Nee**

Als **nee** : .... van de **eerste** en.... van de **tweede partner**

3. Hebt u nog dezelfde partner als toen met de bovenstaande bevalling? **Ja / Nee**

4. Als die bevalling was geëindigd in een keizersnede had u die dan met een

sterilisatie gecombineerd gehad willen hebben**? Ja / Nee** (als **Nee** ga naar **II**)

**I**. **Als Ja:**

Wat typeert uw situatie: (zo nodig **meer dan één antwoord** omcirkelen)

**A**. Ik wil geen kinderen meer en ik vind dat gedoe om een zwangerschap te

voorkomen maar lastig

**B**. Het zou handig geweest zijn om een sterilisatie te combineren met de

keizersnede als die nodig was geweest maar mijn partner en ik hebben weinig

moeite een betrouwbare methode te gebruiken.

**C**. Ik ben bang dat ik nog per vergissing zwanger word.

**D.** Ik zou nu wel een sterilisatie willen maar ik ben wel wat bang voor een operatie

**E.** Ik zou nu wel een sterilisatie willen maar het komt er steeds niet van het te

organiseren.

**F.** Een sterilisatie is te duur.

**G.** Ik vind het dom dat mijn partner en ik niet zelf het initiatief genomen hebben om

een sterilisatie te vragen tegelijkertijd met met een mogelijke keizersnede, het

zou een gemiste kans geweest zijn.

**H.** Ik vroeg om een sterilisatie maar de gynaecoloog wilde niet/raadde het af

**I.** Nu heb ik spijt dat ik niet gesteriliseerd ben, maar toen was ik bang dat mijn baby het niet zou redden dus wilde ik geen drastische besluiten nemen

voor als het een keizersnede was geworden.

**J.** Er was eigenlijk een goede medische reden voor een sterilisatie

**K**. De gynaecoloog sneed het onderwerp sterilisatie in het geval het een keizersnede

zou worden aan, maar deed ook zijn best het direct uit mijn hoofd te praten, dat

liet mij er van afzien

**L.** Anders, nl: .................................................................................... ..........................................................................................................

**II**. **Als Nee:**

Als u ***geen*** sterilisatie had gewild in het geval dat uw laatste bevalling een keizersnede was geweest. Wat typeert dan uw situatie: (zo nodig **meer dan één antwoord** omcirkelen)

A. Ik wil nog meer kinderen, **Hoeveel** ………

B. Ik wil de mogelijkheid openhouden nog meer kinderen te krijgen

C. Ik wil niet meer kinderen maar zou het idee dat ik ze niet meer kan krijgen

vervelend vinden

D. Het was nu mijn partner’s beurt om iets te laten doen *)*

E. Ik heb weinig moeite een andere betrouwbare methode te gebruiken om niet zwanger te worden

F. Mijn religie/cultuur staat een sterilisatie niet toe

G. Als ik 5 jaar ouder was geweest, en die bevalling was een keizersnede

geworden, dan had ik wel een sterilisatie gewild

H. De dokter heeft het niet gevraagd, ik had ook geen sterilisatie gewild maar ik

vind dat hij/zij mij toch had moeten vragen.

I. Ook al had de dokter het mij gevraagd dan had ik zeker niet in verwarring ja

gezegd.

J. Ik wacht tot ik minstens één jongen en één meisje heb voordat ik stop met

kinderen krijgen

K. Andere reden, nl: ........................................................................................

**III**. Kies alstublieft **één** van de onderstaande mogelijkheden die het best bij u past in relatie tot uw  **laatste** niet keizersnede bevalling:

a. De dokter vroeg mij **niet** of ik bij een eventuele nieuwe keizersnede een sterilisatie zou willen. Ik zou **nee** gezegd hebben denk ik. Ik zou daar nu **spijt** van hebben denk ik, als het een keizersnede was geworden, maar ik kon op dat moment niet alles overzien.

b. De dokter vroeg mij **niet** of ik bij een eventuele nieuwe keizersnede een sterilisatie zou willen.Ik zou **nee** gezegd hebben denk ik. Ik zou daar nu **geen spijt** van hebben denk ik, als het een keizersnede was geworden.

c. De dokter vroeg mij **niet** of ik bij een eventuele nieuwe keizersnede een sterilisatie zou willen. Ik zou  **ja** gezegd hebben denk ik. Ik zou daar nu **spijt** van hebben denk ik, als het een keizersnede was geworden, maar ik kon op dat moment niet alles overzien.

d. De dokter vroeg mij **niet** of ik bij een eventuele nieuwe keizersnede een sterilisatie zou willen. Ik zou  **ja** gezegd hebben denk ik. Ik zou daar nu **geen spijt** van hebben denk ik, als het een keizersnede was geworden.

e. De dokter vroeg mij **wel** of ik bij een eventuele nieuwe keizersnede een sterilisatie zou willen. Ik heb **nee** gezegd. Ik zou daar nu **geen spijt** van hebben denk ik, als het een keizersnede was geworden.

f. De dokter vroeg mij **wel** of ik bij een eventuele nieuwe keizersnede een sterilisatie zou willen. Ik heb **nee** gezegd. Ik zou daar nu **spijt** van hebben denk ik, als het een keizersnede was geworden, maar ik kon op dat moment niet alles overzien.

g. De dokter vroeg mij **wel** of ik bij een eventuele nieuwe keizersnede een sterilisatie zou willen. Ik heb **ja** gezegd. Ik zou daar nu **spijt** van hebben denk ik, als het een keizersnede was geworden, maar ik kon op dat moment niet alles overzien.

h. De dokter vroeg mij **wel** of ik bij een eventuele herhaalde keizersnede een sterilisatie zou willen. Ik heb **ja** gezegd. Ik zou daar nu **geen spijt** van hebben denk ik als het een keizersnede was geworden.

5. Een sterilisatie tijdens een keizersnede is eenvoudig. Vindt u dat deze mogelijkheid besproken moet worden met de zwangere en haar partner:

**ja,** want ................................... ................................................................... ...... ............. .................................

**nee**, want............................................................................................................. .

6. Als u de vorige vraag met **ja** beantwoord heeft, vindt u dan dat bespreken voor het eerst gebeuren moet met de keizersnede voor de 2^e^, 3^e^, 4^e^, 5^e^, 6^e^, 7^e^, 8^e^, 9^e^ of 10^e^

baby (Graag juiste **omcirkelen**)

7. Vindt u dat de gemiddelde Nederlandse vrouw, samen met haar partner, in de laatste dagen van de zwangerschap, in staat is om een verantwoorde beslissing te nemen over wel of geen sterilisatie tijdens een keizersnede? ?  **Ja** / **Nee,** evnt. + maar …………………………………………………………………………………………

8. Vindt u dat de verloskundige, gynaecoloog of huisarts er goed aan doet de mogelijkheid van een sterilisatie **in het begin** van de zwangerschap bij vrouwen die al kinderen hebben aan te kaarten? (Zo van: “stel dat u (weer) een keizersnede nodig blijkt te hebben en een stevige baby komt er gezond uit, zou u er vast over willen denken of u dan eventueel een sterilisatie zou willen? “)

Goed om die vraag te stellen **Ja** / **Nee**

9 **A** Wanneer bij uw laatste zwangerschap werd bij u de vraag voor het eerst gesteld door uw **verloskundige / huisarts / gynaecoloog / uzelf/ niemand** of u ook een sterilisatie wilde? (**omcirkel** juiste van deze vijf mogelijkheden)

Was dat

**a** Voor de zwangerschap

**b** Vroeg in de zwangerschap

**c**. Midden zwangerschap **Omcirkel juiste**

**d**. Laatste weken

**e**. Laatste dagen **f.** Laatste uren **g**. Nooit

Hoe heeft u dit ervaren? ……………,

Was dit het juiste moment?.....................................

1. Als u nooit de optie van een sterilisatie aangeboden heeft gekregen, zou u ja

gezegd hebben als het u wel gevraagd was? **Ja / Nee / Weet ik niet**

**Waarom**:.................................................................................................................

1. Als u **wel** de optie van een sterilisatie aangeboden heeft gekregen was dat

een min of meer neutraal aanbod of werd er druk op u uitgeoefend ?

**Neutraal aanbod/ druk om niet / druk om wel gesteriliseerd te worden**

**Licht eventueel toe** .......................................................................................

**D**.Is er druk door uw **omgeving** uitgeoefend om een sterilisatie te krijgen?**Ja/Nee**

**E**.Is er druk door uw **omgeving** uitgeoefend om **geen** sterilisatie te krijgen?**Ja/Nee**

10. Bent u ooit per vergissing zwanger geworden ? **Ja / Nee**

Welk **Jaa**r? ……..

11.Ik vind het niet tot de taak van de dokter behoren het onderwerp anticonceptie

aan te snijden, als ik iets wil, of wil weten neem ik **zelf** het initiatief wel. **Ja / Nee**

12. Wat voor **methode** gebruikt u tegenwoordig om niet zwanger te worden?

(**omcirkel** aub)

**a**.**1.** pil, spiraaltje, ik ben alsnog gesteriliseerd

injectie, kalender methode, inplant

condoom, man gesteriliseerd, zingen en kerk enzo

alleen borstvoeding, geen seks iets anders nl:..................

Als u later gesteriliseerd bent, wanneer was dat**?** 200..,(**jaar** graag), Was dat samen met een bevalling? **Ja / Nee**

**2.** Ik en/of mijn partner maken nogal eens fouten met de methode die we

gebruiken. **Ja / Nee**

**b**. Ik gebruik geen **methode** want:

**1**. Ik denk dat ik/wij niet zwanger kan/kunnen worden,

**2**. Ik heb geen partner

**3**. Ik wil zwanger worden (**omcirkel** juiste(n))

**4**. Ik neem risico’s

**5**. Ik zou het niet zo erg vinden als ik zwanger werd

**6**. Ik ben zwanger **vergissing**/ **geen vergissing**

**7**. Ik denk dat ik te oud word om zwanger te worden

13. Heeft u klachten over de methode die u gebruikt? **Ja / Nee**

Zo ja, **welke:** ...............................................................................................................

...................................................................................................................................... 14. Neem het voorbeeld uit de begeleidende brief van de zwangere dame met 2 kinderen wier derde baby dwars ligt. Er is geen haast en de gynaecoloog bespreekt de mogelijkheid van een sterilisatie met de komende keizersnede **niet**.

**Vindt u dat**: verstandig / een misser / bevoogdend ? (**omcirkel** juiste graag)

15. Neem het voorbeeld uit de begeleidende brief van de zwangere dame met 2 kinderen wier derde baby dwars ligt. Er is geen haast en de gynaecoloog bespreekt de mogelijkheid van een sterilisatie met de komende keizersnede **wel**

**Vindt u dat**: verstandig / een misser / bevoogdend/ bemoeizuchtig? (**omcirkel** juiste graag)

15. Vindt u in het algemeen dat bij een compleet gezin en een man die 2 jaar ouder

is, dat bij een sterilisatie beter **de man of de vrouw** gesteriliseerd kan worden?

16. Hebt u nog opmerkingen/suggesties/klachten ........................................................................................................................... ..........................................................................................

........................................................................................................................... ..........................................................................................

**Heel hartelijk bedankt voor uw medewerking**

Vakgroep gynaecologen Hoogeveen

(evnt **uw e-mail adres**  ………… @..........................

(voor als u een verslag van de uitkomst van deze studie wilt)
