# Supplementary material for: The Right to Informed Choice. A Study and Opinion Poll of Women Who Were or Were Not Given the Option of a Sterilisation with Their Caesarean Section
Source: PLoS One. 2011 Mar 22;6(3):e14776. doi: 10.1371/journal.pone.0014776 (PMC3062542; doi:10.1371/journal.pone.0014776)
Supplement: Supporting Information S7 — Questionnaire vaginal delivery, CS earlier. (0.05 MB DOC) [file pone.0014776.s007.doc]

**Questionnaire for women who have had a Caesarean Section (CS) earlier**

Regarding the vaginal delivery on - -

**Please circle or fill in the appropriate answer(s).**

1. How many living children do you have in total? …… **Boys**, …… **Girls.**

2. Are all your children from the same partner? **Yes/No**

If not: ……. from the first and ……. from the second partner.

3. Do you still have the same partner as you did at the above delivery? **Yes/No**

4. If that delivery had ended in a Caesarean section (CS), would you have liked it to be combined with a sterilisation? **Yes/No** (if you answered **No,** go straight to **II**)

**I**. **If you answered Yes:** What characterises your situation/opinion:
 (You may circle more than one answer if necessary)

**A.** I do not want to have more children and feel burdened by having to prevent falling pregnant.

**B.** It would have been convenient to have had also a sterilisation if it would have been a Caesarean   delivery, but my partner and I have little trouble using reliable contraception.
 **C.** I worry that I will become unintendedly pregnant one of these days.
 **D.** I would like a sterilisation now but am afraid of the operation.
 **E.** I would like a sterilisation now but procrastinate organising it.
 **F.** A sterilisation I could organise now is too expensive for me.
 **G.** I think my partner and I lacked foresight when we didn’t take the initiative to get a sterilisation   with a possible CS. It would have been a missed opportunity.
 **H.** I asked for a sterilisation but the obstetrician advised against it/ refused.
 **I.** I now regret that I did not have the opportunity to be sterilised, but at the time of delivery I was   afraid something could happen to my baby and did not want to make an irreversible decision.
 **J.** There was actually a good medical reason for a sterilisation.
 **K.** The obstetrician raised the subject of having a sterilisation with a possible CS but   dissuaded me at the same time. This decided me against having a sterilisation.
 **L.** Other: ..……………………………………………………………………..……………..
 ……………………………………………………………………….………………………..

**II**. If you had **not** wanted a sterilisation in the case that your last delivery was a CS, please circle **all** the answers that characterise your situation/opinion:

**A.**  I want more children. How many? ………

**B.** I want the option of having more children.

**C.** I don’t want more children, but do not like the idea of not being able to have more.

**D.** It was my partner’s turn to have something done.

**E.** I have little trouble using a reliable method to prevent a pregnancy.

**F.** My religion/culture does not allow sterilisation without a good medical reason.

**G.** If I had been 5 years older and that delivery had been a CS, I would have liked a     sterilisation.

**H.** The doctor did not ask me. I would not have wanted a sterilisation but I think that he/she should    have asked me anyway.

**I.**  Even if the doctor had asked me I would definitely not have said yes in the confusion.

**J.** I am waiting until I have at least one boy and one girl before I stop having children.

**K.**  Other:…………...............................................................................................................................

….........................................................................................................................................................

**III**. Please choose **one** of the following options that best applies to your situation in relation to your **last** delivery (options continue on the following page):

**A.** The doctor did **not** ask me whether I would want a sterilisation at a potential next CS. I think I would have said **no**. If I had delivered by CS this time, I think I would have **regretted** that decision, but I couldn’t oversee everything at the time.

**B.** The doctor did **not** ask me whether I would want a sterilisation at a potential next CS. I think I would have said **no.** If I had delivered by CS this time, I would **not have regretted** that decision.

**C.** The doctor did **not** ask me whether I would want a sterilisation at a potential next CS. I think I would have said **yes.**  I think I would **regret** that decision now if I had delivered by CS, but I couldn’t oversee everything at the time.

**D.** The doctor did **not** ask me whether I would want a sterilisation at a potential next CS. I think I would have said **yes.** I think I **would not regret** this decision if I had delivered by CS this time.

**E.** The doctor **did** ask me whether I would want a sterilisation at a potential next CS. I said **no.** I think I would **not** **regret** that decision now if I had delivered by CS this time.

**F.** The doctor **did** ask me whether I would want a sterilisation at a potential next CS. I said **no**. I think I would have **regretted** that decision now if I had delivered by CS, but I could not oversee everything at the time.

**G.** The doctor **did** ask me whether I would want a TO at a potential next CS. I said **yes**. I think I would **regret** that decision now if I had delivered by CS, but I could not oversee everything at that moment.

**H.** The doctor **did** ask me whether I would want a sterilisation at a potential next CS. I said **yes**. I think I would **not regret** that decision now if I had delivered by CS last time.

**I.** Other: ………………………………………………………………………………………………..
……………………………………………………………………………………………………..........

5. Performing a sterilisation during a CS is easy. Do you think this option should be discussed with a pregnant woman and her partner? **Yes/No**

**Because**…………………………………………………………………………………………………………………….……………………………………………………………………………………………………………………………………..

6. If you answered the previous question with **Yes**, do you think this should be discussed for the first time before the CS for the 2nd, 3rd, 4th, 5th, 6th, 7th, or 8th child?
(Circle the number you prefer)

7. Do you think that the average Dutch woman is able, together with her partner, **in the last days of her pregnancy**, to make a responsible decision about whether to have a sterilisation combined with her CS?  **Yes**/**No**, but **(optional)**………..……………………………………………..……………………………………….
……………………………………………………………………………………………....................

8. Are you of the opinion that a midwife, obstetrician or GP should discuss **early during pregnancy** the option of sterilisation with women who already have children? (Something like: *“Suppose you happen to need a CS (again) and a healthy, strong baby is delivered, could you please consider in the months to come whether you would also like a sterilisation?*)

Is such a question appropriate? **Yes**/**No**

9. **A.** In what phase of your last pregnancy did your **midwife/GP/gynaecologist/yourself/nobody** first ask whether you also wanted to be sterilised? (Please **circle** one of these five options)

Was that:

**a.** Before the pregnancy

**b.** Early on in the pregnancy

**c**. Mid-pregnancy

**d**. In the last weeks

**e**. In the last days

**f**. In the last hours

**g**. Never

How did you feel about this? …………………………………………………………….….…………...

Was it the right moment? .......................................................................................................................

1. **If you were never offered the option of a sterilisation: would you have said yes if you had been** offered one? **Yes/No/Don’t know**

**Why**:.................................................................................................................

1. If you **were** offered the option of sterilisation: was this a more or less neutral offer, or did you feel pressured to make a certain decision?

**Neutral offer/ pressure to get sterilised/pressure to not get sterilised**

Room to elaborate (optional)................................................................................................................
……………………………………………………………………………………………………………

**D.** Did your environment make you feel pressured to get a sterilisation? **Yes/No
E**. Did your environment make you feel pressured to **not** get a sterilisation? **Yes/No**

10. Have you ever become pregnant unintendedly? **Yes/No**

In what year? ……..

11. I am of the opinion that a doctor should **not** raise the subject of contraception. If I want something or want to know something, I will take the initiative myself: **Yes/No.**

12. **A**.**1.** What method are you presently using to prevent pregnancy? (**Please circle**)

contraceptive pill intra uterine device (coil) abstinence contraceptive injection fertility awareness (calendar) implant

condom partner had a vasectomy withdrawal

just lactation I have since had a sterilisation other: ...................

If you were sterilised at a later stage in another hospital when was that? 200… (**year**).
Was that in combination with a delivery? **Yes/No**

**2.** Me and/or my partner quite often make mistakes with the method that we are using**. Yes/No**

**B.** I don’t use **any** method because:

**1**. I don’t think that I/we can become pregnant

**2**. I don’t have a partner

**3**. I want to become pregnant (**circle the appropriate answer(s))** **4**. I take risks

**5**. I wouldn’t mind becoming pregnant

**6**. I am pregnant (**a mistake/not a mistake)**

**7**. I think I am becoming too old to become pregnant

13. Do you have any complaints about the method you use? **Yes/No**

If **yes**, what complaints? .......................................................................................................................

............................................................................................................................................................. .............................................................................................................................................................

**In the questions below, please circle the answers that best reflect your opinion.**

14. Consider the example of the enclosed letter involving a woman with 2 children whose third is lying in a transverse position. There is no hurry and the obstetrician **does not** counsel her about the option of a sterilisation with the coming CS. 

Do you find that: **sensible/a mistake/patronising**

15. Consider the example from the enclosed letter involving a woman with 2 children whose third is lying in a transverse position. There is no hurry and the obstetrician **does** counsel her about the option of a sterilisation with the coming CS.

Do you find that: **sensible/a mistake/patronising/meddlesome**

16. Generally speaking, do you think that in a complete family, with a man who is two years older than the woman, it is better that **the man** or **the woman** gets sterilised?

17. Do you have other remarks/suggestions/complaints? ..............................................................................................................................................................................................................................................................................................................................

..............................................................................................................................................................................................................................................................................................................................

**Thank you very much for your cooperation**

Obstetricians, Hoogeveen.

Optional: write down your e-mail address if you want to see the results of this study:

…………………………@………………………………
